# Synthesis of Saccharumoside-B analogue with potential of antiproliferative and pro-apoptotic activities

**DOI:** 10.1038/s41598-017-05832-w

**Published:** 2017-08-16

**Authors:** Srinuvasarao Rayavarapu, Nagendra Sastry Yarla, Sunanda Kumari Kadiri, Anupam Bishayee, Siddaiah Vidavalur, Ramu Tadikonda, Mahaboob Basha, Vijaya Rao Pidugu, Kaladhar S. V. G. K. Dowluru, Dhananjaya Bhadrapura Lakappa, Mohammad A. Kamal, Ghulam Md Ashraf, Vadim V. Tarasov, Vladimir N. Chubarev, Sergey G. Klochkov, George E. Barreto, Sergey O. Bachurin, Gjumrakch Aliev

**Affiliations:** 1 Department of Organic Chemistry, Foods, Drugs and Water, College of Science and Technology, Andhra University, Visakhapatnam, 530 003 Andhra Pradesh India; 2 Department of Biochemistry and Bioinformatics, School of Life Sciences, Institute of Science, GITAM University, Visakhapatnam, 530 045 Andhra Pradesh India; 3 Department of Animal Biology, University of Hyderabad, Hyderabad, 500 046 Telangana India; 4 Department of Microbiology, College of Science and Technology, Andhra University, Visakhapatnam, 530 003 Andhra Pradesh India; 5 Department of Pharmaceutical Sciences, College of Pharmacy, Larkin Health Sciences Institute, Miami, FL 33169 USA; 6 Excelra Knowledge Solutions Private Limited, NSL SEZ ARENA, IDA Uppal, Hyderabad, 500 039 Telangana India; 7 Department of Microbiology and Bioinformatics, Bilaspur University, Bilaspur, 495 001 Chhattisgarh India; 8 Toxinology/Toxicology and Drug Discovery Unit, Center for Emerging Technologies, Jain Global Campus, Jain University, Kanakapura Taluk, Ramanagara, 562 112 Karnataka India; 9 Enzymoics and Novel Global Community Educational Foundation, Hebersham, NSW Australia; 10 King Fahd Medical Research Center, King Abdulaziz University, Jeddah, Saudi Arabia; 11 Institute of Pharmacy and Translational Medicine, Sechenov First Moscow State Medical University, 119991 Moscow, Russia; 12 Institute of Physiologically Active Compounds of the Russian Academy of Sciences, Severniy Proezd, Chernogolovka, Moscow Region 1142432 Russia; 13 Departamento de Nutrición y Bioquímica, Facultad de Ciencias, Pontificia Universidad Javeriana, Bogotá, D. C. Colombia; 14 Instituto de Ciencias Biomédicas, Universidad Autónoma de Chile, Santiago, Chile; 15 “GALLY” International Biomedical Research Consulting LLC, San Antonio, TX 78229 USA; 16 School of Health Sciences and Healthcare Administration, University of Atlanta, Johns Creek, GA 30097 USA

**Keywords:** Cancer, Breast cancer

## Abstract

A new series of phenolic glycoside esters, saccharumoside-B and its analogs (**9b**-**9n**, **10**) have been synthesized by the Koenigs-Knorr reaction. Antiproliferative activities of the compounds (**9b**-**9n**, **10**) were evaluated on various cancer cell lines including, MCF-7 breast, HL-60 leukemia, MIA PaCa-2 pancreatic, DU145 prostate, HeLa cervical and CaCo-2 colon, as well as normal human MCF10A mammary epithelial and human peripheral blood mononuclear cells (PBMC) by MTT assay. Compounds (**9b**-**9n**, **10**) exhibited considerable antiproliferative effects against cancer cells with IC_50_ range of 4.43 ± 0.35 to 49.63 ± 3.59 µM, but they are less cytotoxic on normal cells (IC_50_ > 100 µM). Among all the compounds, **9f** showed substantial antiproliferative activity against MCF-7 and HL-60 cells with IC_50_ of 6.13 ± 0.64 and 4.43 ± 0.35, respectively. Further mechanistic studies of **9f** were carried out on MCF-7 and HL-60 cell lines. **9f** caused arrest of cell cycle of MCF-7 and HL-60 cells at G0/G1 phase. Apoptotic population elevation, mitochondrial membrane potential loss, increase of cytosolic cytochrome *c* and Bax levels, decrease of Bcl-2 levels and enhanced caspases-9 and -3 activities were observed in **9f**-treated MCF-7 and HL-60 cells. These results demonstrate anticancer and apoptosis-inducing potentials of **9f** in MCF-7 and HL-60 cells via intrinsic pathway.

## Introduction

Cancer has become the major cause of death in the world with the changes in the living habitat of people and environment^1, 2^. Breast cancer is the leading cause of cancer deaths in women with an estimated 1,383,500 new cases and 458,400 deaths annually^3, 4^. Chemotherapy is one of the treatments for cancer. Hence, chemotherapeutic agents have been developed throughout the world by pharmaceutical industries and academic institutions but their usage is limited due to poor efficacy and adverse effects^5–7^. Drug resistance is also another problem in cancer treatment^8, 9^. Thus, discovery and development of safer and effective molecules are urgently required to reduce the burden of cancer.

Natural products have been isolated from natural sources and explored their use as drugs for various diseases including cancers. However, there are many challenges in discovery and development of natural products as drugs. Yields, time-consuming isolation processes and impurities are some of the major challenges in isolation and identification of natural products^10^. Bioactivity studies including *in vitro*, preclinical and clinical, require high quantities of compounds for evaluating their bioactivities^10^. Some of the natural products have been found with moderate bioactivities and poor pharmacokinetic properties^11^. To overcome these problems, natural products have been synthesized chemically as in their native form and/or their analogs and explored their utility as drugs^11–13^. For instance, dried leaves from almost 15 trees of *Vinca rosea* (*Catharanthus roseus*) required to obtain only 30 g of vincristine (a chemotherapeutic drug). Similarly, 27,300 kg of the bark of *Taxus brevifolia* required to obtain 1900 g of taxol. Later, vincristine and taxol have been synthesized in high quantities by several total and semi-synthetic methods^10^. Discodermolide, a marine-derived anticancer agent from marine sponge *Discodermia dissoluta* was not available in sufficient quantities to carry out clinical trials^10^. Later, discodermolide was synthesized with high yields and used in clinical trials for cancers^14, 15^.

Phenolic glycoside esters (PG) are some of the most abundant secondary metabolites in plants with novel bioactivities^16^. Several PG have been isolated from the plants of the different families and reported to possess various bioactivities including anticancer activity^17–19^. Previously, Tao *et al*.^19^ isolated four new PG esters including saccharumoside B from the bark of *Acer saccharum* (sugar maple tree) and reported cytotoxic activity of saccharumoside B on human colon cancer cell line. Mechanism of action of cytotoxic saccharumoside B in cancers remains to be elusive. The present work reports for the first time the synthesis of anticancer natural product saccharumoside B and its analogs with good yields. Further, *in vitro* anticancer and pro-apoptotic studies of synthesized saccharumoside-B and its analogs were performed.

## Results

### Chemistry

**Figure 1:**
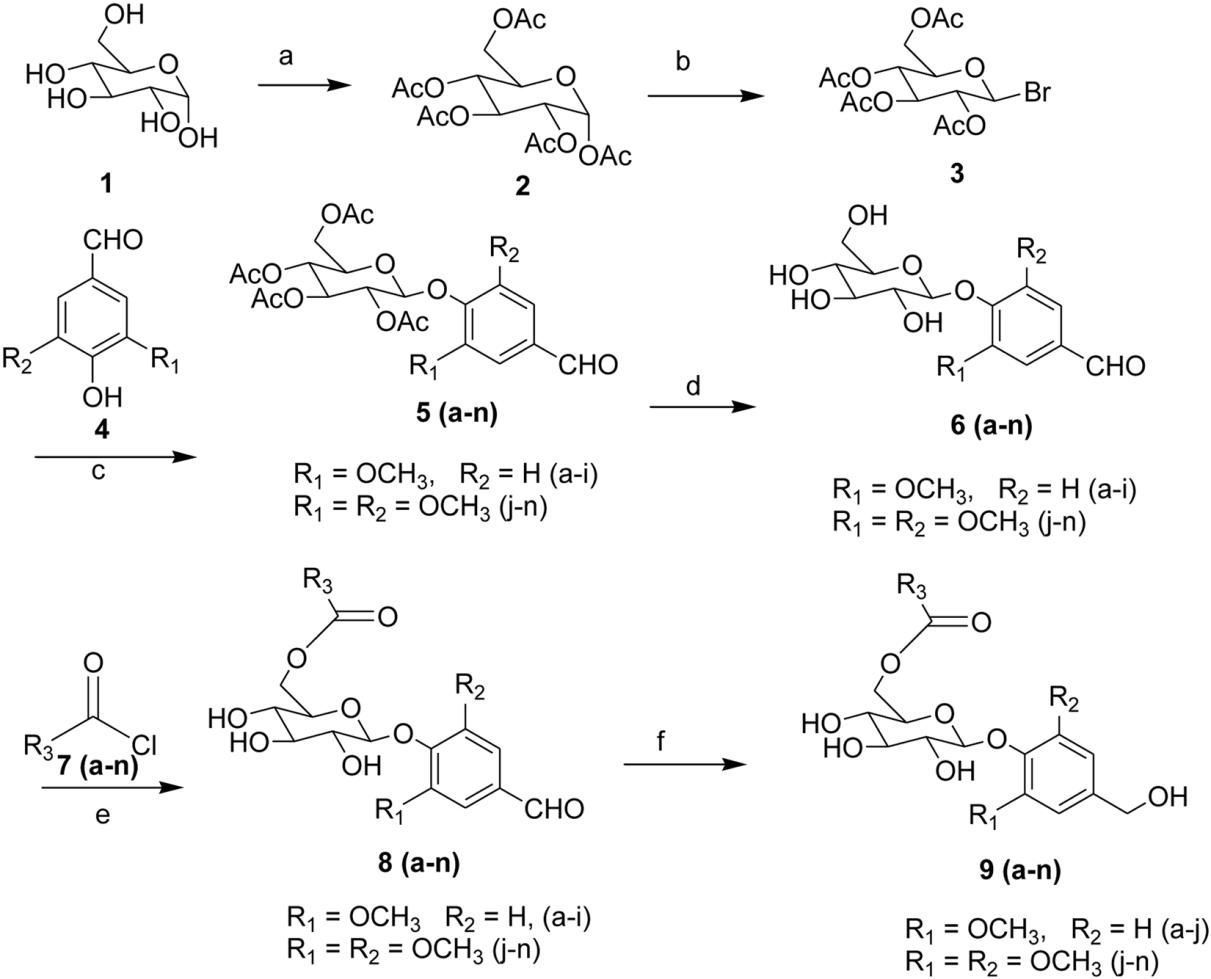
Synthesis of saccharumoside-B analogs. Reagents and conditions: (**a**) Ac_2_O/AcOH, HClO_4_ in Ac_2_O; (**b**) HBr in glacial acetic acid; (**c**) K_2_CO_3_, DCM-H_2_O, aliquat-336; (**d**) NaOMe, MeOH; (**e**) Pyridine, DCM; (**f**) NaBH_4_, MeOH.

**Figure 2:**
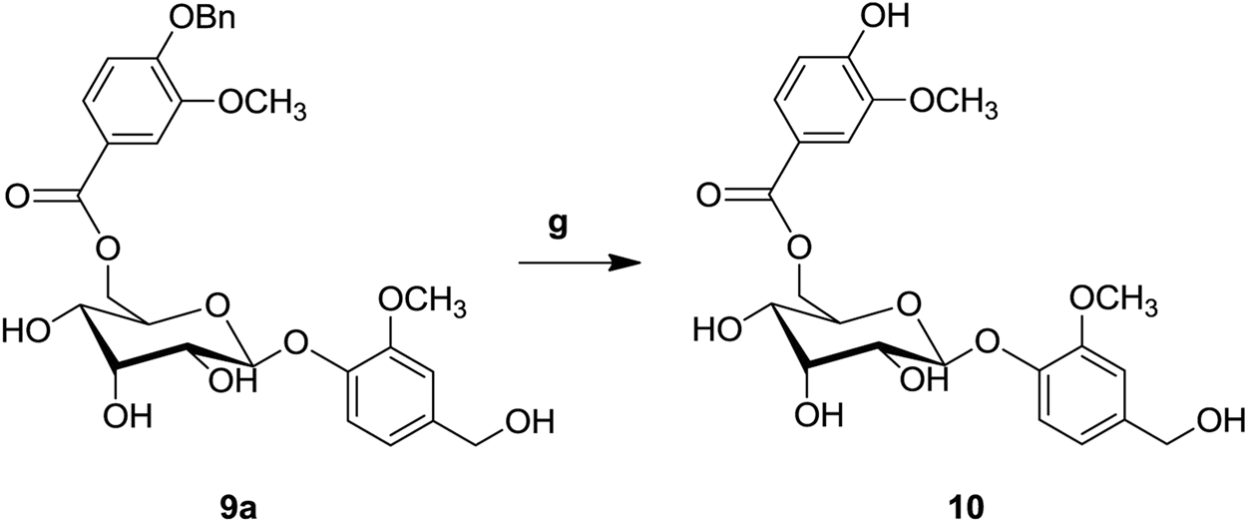
Synthesis of saccharumoside-B. Reagents and conditions: (**g**) Pd/CaCO_3_, H_2_ gas, TEA.

**Figure 3:**
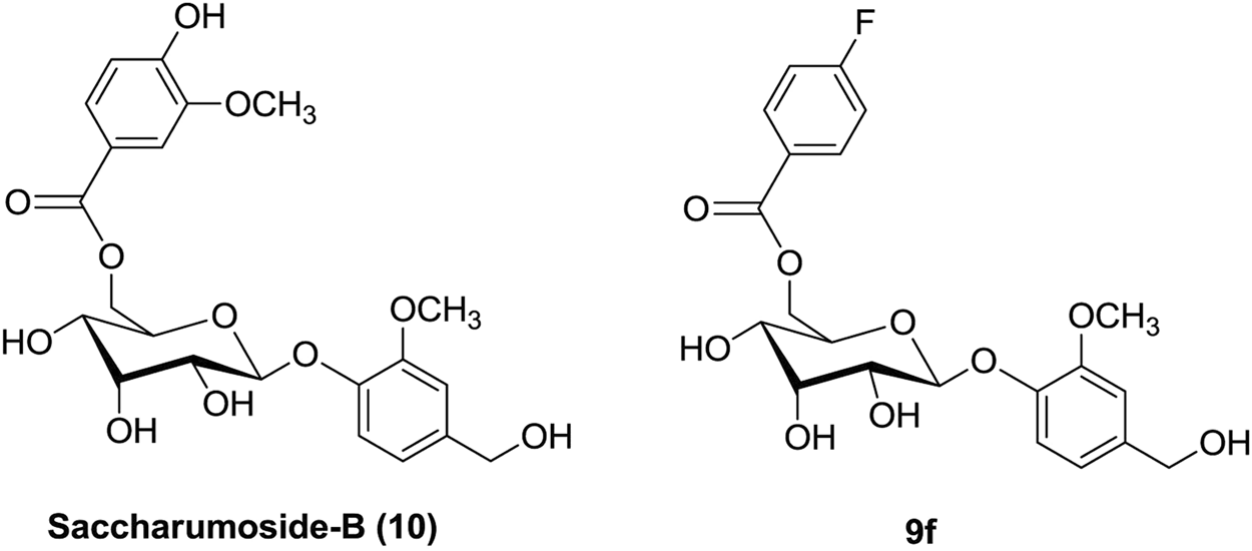
Structures of saccharumoside-B and its analog **9f**.

Synthesis of saccharumoside-B and its analogs (Table 1) was performed according to the pathway illustrated in Figs 1 and 2. First, α-D-glucose (**1**) was treated with Ac_2_O/AcOH in presence of HClO_4_ in Ac_2_O to provide pentaacetyl-α-D-glucose (**2**). Subsequently, compound **2** was treated with HBr in glacial acetic acid to get α-D-acetobromo glucose (**3**). Different phenolic glycoside derivatives (**5a-n**) were prepared by the reaction of compound **3** with different 4-hydroxy benzaldehydes (**4a-n**) using K_2_CO_3_, aliquat-336 and CH_2_Cl_2_:H_2_O (1:1). Deacylation of **5a-n** was carried out in presence of NaOMe in MeOH to afford corresponding products **6a-n**. Benzoylation of **6a-n** using different benzoyl chlorides (**7a-n**) and pyridine led to the formation of a mixture of isomers, which were further separated by using silica gel column chromatography (100–200 mesh) to get pure compounds **8a-n**. The aldehyde group of **8a-n** was reduced by employing NaBH_4_ in MeOH to give compounds **9a-n**. Finally, the synthesis of naturally occurring phenolic glycoside ester, saccharumoside-B (**10**) (Fig. 3) was done by the debenzylation of compound **9a** using Pd/CaCO_3_ and TEA under H_2_ atmosphere. Spectral data of synthetic sacharramoside-B was found to be identical to those of reported isolated product (Supplementary Figures 1–28)^19^.

**Table 1:** Synthesis of saccharumoside-B and its analogs.

**S**.**No.**	**Entry**	**R**_**1**_	**R**_**2**_	**R**_**3**_	**Yield** (**%**)^**a**^	**M**.**P** (^**o**^**C**)
1	**10**	OCH_3_	H		40	200–202
2	**9b**	OCH_3_	H		47	187–189
3	**9c**	OCH_3_	H		45	228–230
4	**9d**	OCH_3_	H		48	199–201
5	**9e**	OCH_3_	H		50	168–170
6	**9f**	OCH_3_	H		30	209–211
7	**9g**	OCH_3_	H		45	128–130
8	**9h**	OCH_3_	H		31	194–196
9	**9i**	OCH_3_	H		30	186–188
10	**9j**	OCH_3_	OCH_3_		48	180–182
11	**9k**	OCH_3_	OCH_3_		45	201–203
12	**9l**	OCH_3_	OCH_3_		47	131–133
13	**9m**	OCH_3_	OCH_3_		30	170–172
14	**9n**	OCH_3_	OCH_3_		43	124–126

^a^Isolated yield.

### Antiproliferative activities of saccharumoside-B and its analogs

**Figure 4:**
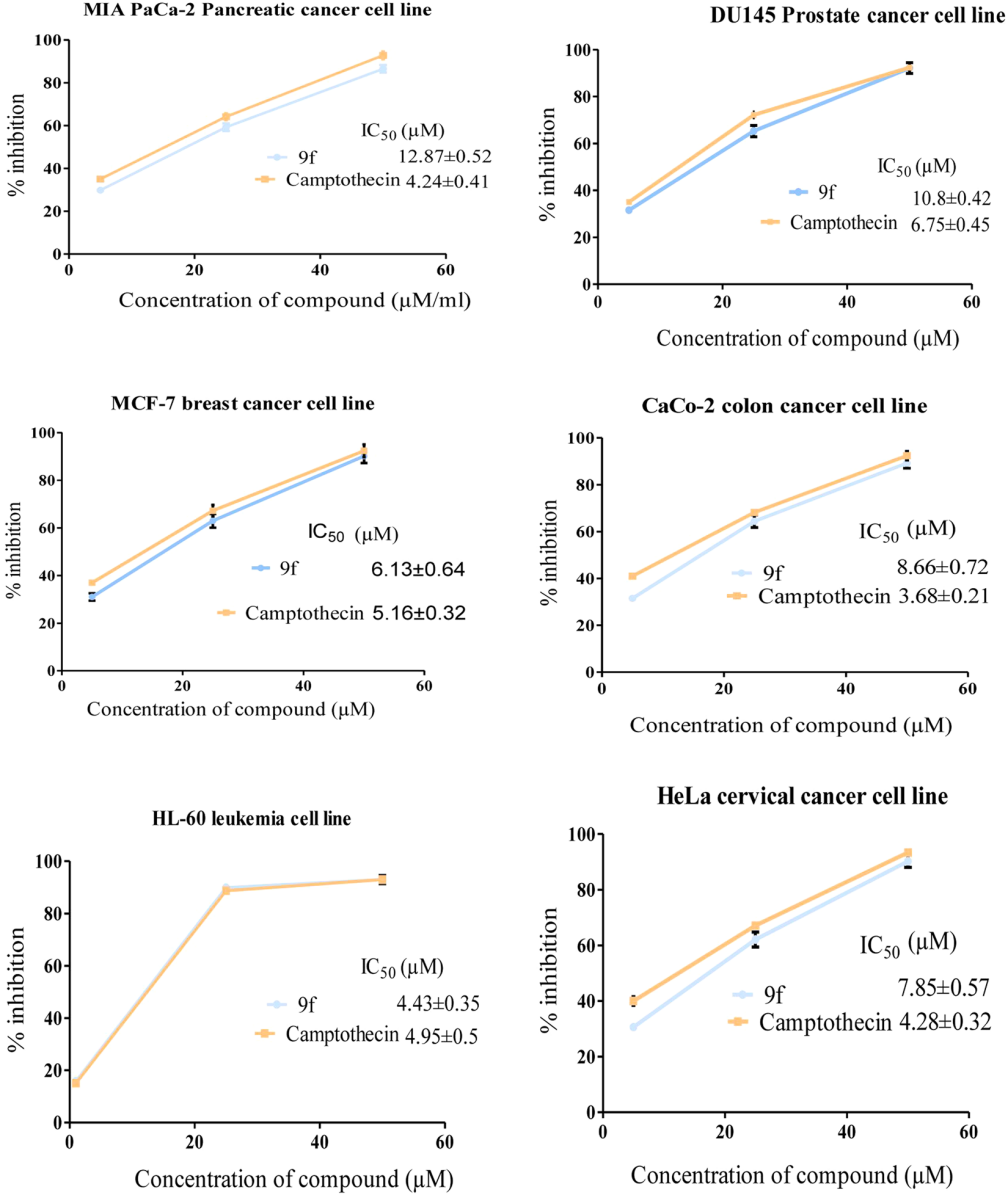
Antiproliferative activity of 9f on cancer cell lines. Antiproliferative effect of 9f on Mia PaCa-2 pancreatic, DU145 prostate, MCF-7 breast CaCo-2 colon cancer, HL-60 leukemia and HeLa cervical cancer cell lines is presented in dose-dependent manner. IC_50_ values of the **9f** and camptothecin (positive control) are depicted in the figure. Data are presented as the mean ± SEM of three independent experiments.

Anticancer activity of synthesized phenolic glycoside esters (**9b**-**9n**, **10**) was tested on MCF-7 breast, MIA PaCa-2 pancreatic, DU145 prostate, CaCo-2 colon, HL-60 leukemia and HeLa cervical cancer cells as well as on normal MCF10A mammary epithelial cells and PBMCs by MTT assay. As shown in Table 2, all compounds (**9b**-**9n**, **10**) showed considerable antiproliferative activity against cancer cells with IC_50_ range of 4.43 ± 0.35 to 49.63 ± 3.59 µM. Whereas, the compounds (**9b**-**9n**, **10**) showed less cytotoxicity against non-cancerous MCF-10A and PBMC cells (IC_50_ > 100 µM) (Supplementary Figure 29). Among all compounds, **9f** showed substantial antiproliferative effect on Mia PaCa-2 pancreatic, DU145 prostate, MCF-7 breast, CaCo-2 colon, HL-60 leukemia and HeLa cervical cancer cell lines with IC_50_ of 12.87 ± 0.52, 10.8 ± 0.42, 6.13 ± 0.64, 8.66 ± 0.72, 4.43 ± 0.35 and 7.85 ± 0.57 µM, respectively, whereas camptothecin (positive control) exhibited antiproliferative activity with IC_50_ of 4.24 ± 0.41, 6.75 ± 0.45, 5.16 ± 0.32, 3.68 ± 0.21, 4.95 ± 0.5 and 4.28 ± 0.32 µM, on respective cancer cell lines (Fig. 4). Fluoro substituent on the phenyl ring of ester group imparts enhanced anticancer activity on various cancer cells to compound **9f** when compared to saccharumoside-B (Fig. 3). As significant inhibition of MCF-7 and HL-60 cells by **9f** was observed, further experiments were aimed to demonstrate the mechanistic insights of **9f** in MCF-7 breast and HL-60 leukemia cancer cells.

**Table 2:** Antiproliferative activities of saccharumoside-B and its analogs against various cancer and normal cell lines.

S.No.	IC_50_ (µM)^a^							
	Cancer cell lines						Normal cell line	
	MIA PaCa-2 Pancreatic	DU145 Prostate	MCF-7 Breast	CaCo-2 Colon	HL-60 Leukemia	HeLa Cervical	MCF-10A Breast	PBMC
**10**	28.32 ± 0.76	33.46 ± 1.43	24.90 ± 1.67	44.67 ± 2.52	31.78 ± 1.82	39.87 ± 2.6	>100	>100
**9b**	39.34 ± 1.42	37.34 ± 2.54	26.43 ± 0.96	47.34 ± 1.62	22.75 ± 1.3	36.68 ± 2.31	>100	>100
**9c**	13.34 ± 0.25	12.36 ± 0.47	9.34 ± 0.48	13.67 ± 0.63	12.45 ± 0.81	14.22 ± 0.68	>100	>100
**9d**	44.56 ± 2.67	42.45 ± 1.51	28.34 ± 1.47	37.35 ± 1.65	25.21 ± 1.92	39.5 ± 2.43	>100	>100
**9e**	36.34 ± 1.58	49.39 ± 1.72	33.37 ± 0.76	43.45 ± 1.87	22.15 ± 1.32	41.52 ± 2.1 8	>100	>100
**9 f**	12.87 ± 0.52	10.8 ± 0.42	6.13 ± 0.64	8.66 ± 0.72	4.43 ± 0.35	7.85 ± 0.57	>100	>100
**9 g**	23.45 ± 1.53	38.32 ± 0.37	28.85 ± 0.47	43.87 ± 2.52	33.52 ± 1.45	41.92 ± 3.15	>100	>100
**9 h**	33.87 ± 1.41	42.11 ± 1.52	33.85 ± 0.48	49.04 ± 0.48	30.23 ± 1.32	35.36 ± 2.2	>100	>100
**9i**	34.78 ± 1.93	43.56 ± 1.87	34.78 ± 0.51	46.98 ± 2.58	29.62 ± 0.83	38.36 ± 1.53	>100	>100
**9j**	45.76 ± 0.42	36.78 ± 0.59	29.45 ± 0.79	33.56 ± 1.51	25.57 ± 0.94	30.43 ± 2.81	>100	>100
**9k**	23.87 ± 0.74	42.78 ± 0.59	21.97 ± 0.72	28.26 ± 1.47	18.53 ± 0.78	26.55 ± 1.37	>100	>100
**9 l**	43.76 ± 1.75	49.63 ± 3.59	33.84 ± 0.73	41.11 ± 1.48	31.77 ± 1.58	36.28 ± 1.76	>100	>100
**9 m**	47.34 ± 2.54	33.78 ± 1.27	28.43 ± 0.69	35.56 ± 1.46	21.82 ± 1.53	34.15 ± 1.83	>100	>100
**9n**	26.78 ± 1.25	37.89 ± 2.48	34.95 ± 1.52	45.23 ± 1.79	28.34 ± 1.72	32.11 ± 1.35	>100	>100
Camptothecin	4.24 ± 0.41	6.75 ± 0.45	5.16 ± 0.32	3.68 ± 0.21	4.95 ± 0.5	4.28 ± 0.32	>100	>100

^a^Data represented as mean of the three independent experiments ± S.E.M.

### Apoptosis inducing activity of 9f on MCF-7 breast cancer cell line

**Figure 5:**
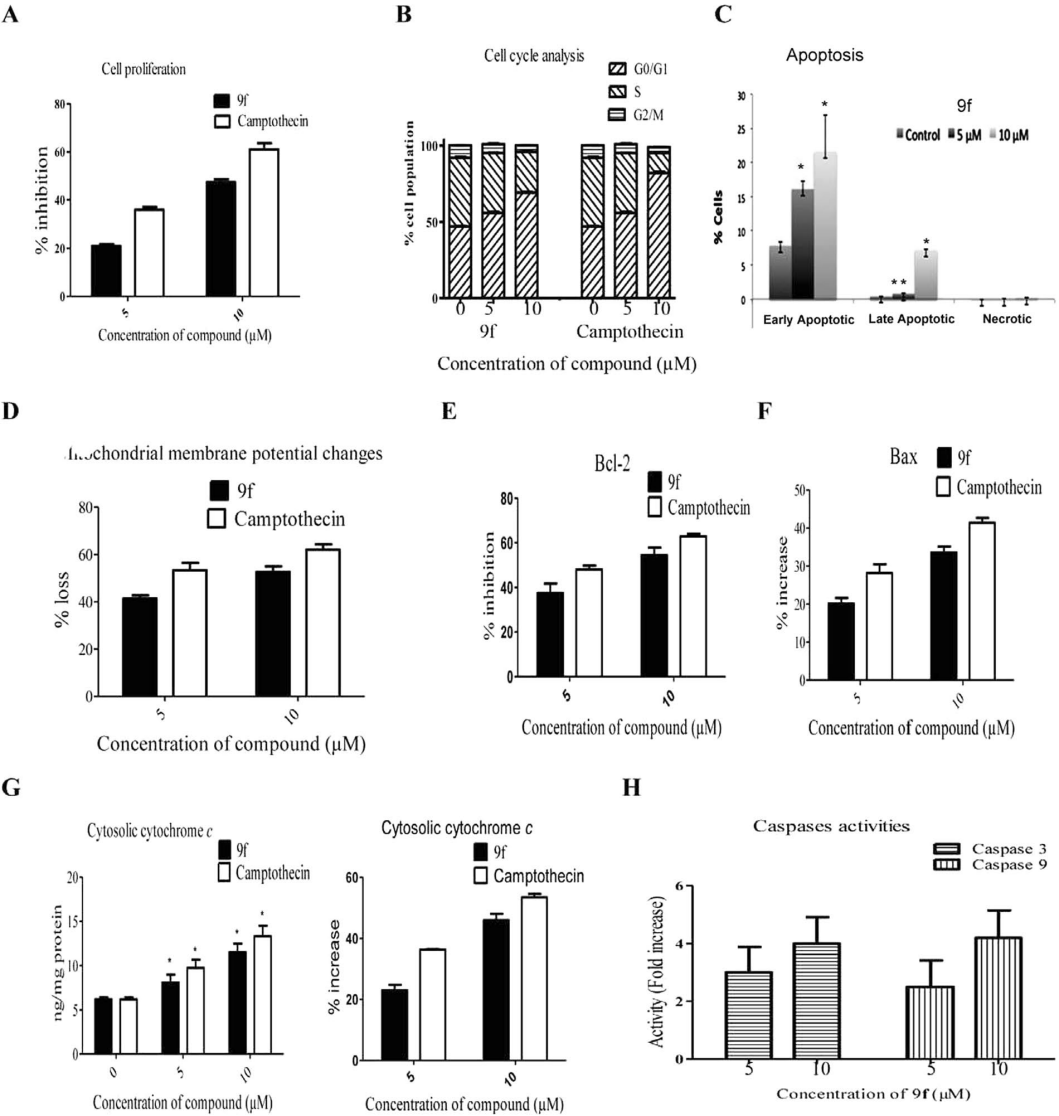
Apoptosis-inducing activity of 9f in MCF-7 breast cancer cells. Panel A: Antiproliferative effect of **9f** (5 and 10 µM) in MCF-7 at 24 h. Panel B: Effect of **9f** on cell cycle distribution of MCF-7 cancer cells. Panel C: Apoptotic cell population in **9f** treated MCF-7 cells. Panel D: Effect of **9f** on mitochondrial membrane potential in MCF-7 cells. Panel E: Effect of **9f** on Bcl-2 levels represented in percent inhibition in treated and untreated MCF-7 cancer cells. Panel F: Distribution of Bax levels represented in percent increase in **9f** treated MCF-7 cancer cells. Panel G: Effect of **9f** in cytosolic cytochrome *c* levels represented in ng/ml and percent increase in treated and untreated MCF-7 cells. Panel H: Effect of **9f** on caspases 3 and 9 activities represented in fold increase in treated MCF-7 cancer cells as compared to untreated. Results of apoptosis-inducing activity of **9f** are compared with camptothecin (positive control). Each bar represents mean ± SEM of three independent experiments; *P < 0.05, **P < 0.01 *versus* untreated control.

MCF-7 cancer cells (1 × 10^6^/ml) were exposed to compound **9f** at 5 and 10 µM concentrations for 24 h and used for cell proliferation and pro-apoptotic studies (Fig. 5). Antiproliferative effect of 9f against HL-60 cells at 24 h was presented in Panel A of Fig. 5. Cell cycle distribution of MCF-7 cancer cells was analyzed by flow cytometry. The cells were stained with propidium iodide (PI) and analyzed by flow cytometry to determine the distribution of the cell population in different phases (G0/G1, S, and G2/M) of cell cycle. Camptothecin-treated cells represented as positive control. Panel B of Fig. 5 represents the percent distribution of MCF-7 cancer cells in each stage of the cell cycle following incubation with **9f** for a period of 24 h. **9f-**treated MCF-7 cells resulted in considerable accumulation of cells in the G0/G1 phase of cell cycle with a concomitant decrease in the number of cells in both the S and G2/M phases in a dose-dependent manner. This demonstrates the cell cycle arrest at G0/G1 phase (Supplementary Figure 30). As shown in Panel C of Fig. 5, apoptotic cell death of MCF-7 breast cancer cells by **9f** was quantified by flow cytometry using Annexin V-PI staining. The results demonstrated that **9f** caused a dose-dependent increase in the early apoptotic to late apoptotic population in MCF-7 cells (Panel C of Fig. 5; Supplementary Figure 31).

Mitochondrial membrane potential loss (Δψm), an indicator of mitochondria membrane disruption, is a distinctive feature of the initial stages of apoptosis. In rhodamine-123 based mitochondrial membrane potential (Δψm) assay, **9f** caused 41.33 ± 1.43% and 52.67 ± 2.33% mitochondria membrane potential loss in MCF-7 cells at 5 and 10 µM concentrations, respectively. Similarly, camptothecin (positive control) caused 53.33 ± 3.06% and 62 ± 2.31% mitochondrion membrane potential loss in MCF-7 cells at 5 and 10 µM concentrations, respectively, (Fig. 5 panel D). Bcl-2 and Bax expression levels are associated with integrity of mitochondria membrane and involved in the regulation of apoptosis. Compound **9f** showed dose dependent inhibition of Bcl-2 in treated MCF-7 breast cancer cells as compared to untreated cells (Fig. 5, panel E). Bax protein levels are dose dependently increased in treated MCF-7 cancer cells compared to untreated cells (Fig. 5, panel F). Overall, these results demonstrate the decreasing the ratio of Bcl-2/Bax in **9f**-treated MCF-7 cancer cells (Fig. 5). This is one of the indications of apoptosis. Cytosolic cytochrome *c* levels elevation is a hallmark of apoptosis. To determine the release of cytochrome *c* from mitochondria into cytosol, cytosolic fractions were collected from the MCF-7 cells treated with compound **9f** (5, 10 µM), and subjected to antibody coated ELISA based protein level estimation. Cytosolic cytochrome *c* levels are dose dependently increased in treated MCF-7 cancer cells (Fig. 5, panel G). Caspases activation is a key process in apoptosis. Caspases-3, -8 and -9 colorimetric assays were performed to estimate the level of caspases (3, 8 and 9) activation before and after treatment with **9f**. Exposure of cancer cells to **9f** enhanced caspase-3 and -9 activities (Fig. 5, panel H), but it did not show considerable effect on caspase-8 activity (data not showed). These results demonstrated the pro-apoptotic potential of **9f** in MCF-7 cells via mitochondria-mediated pathway.

### Apoptosis inducing activity of 9f on HL-60 leukemia cell line

**Figure 6:**
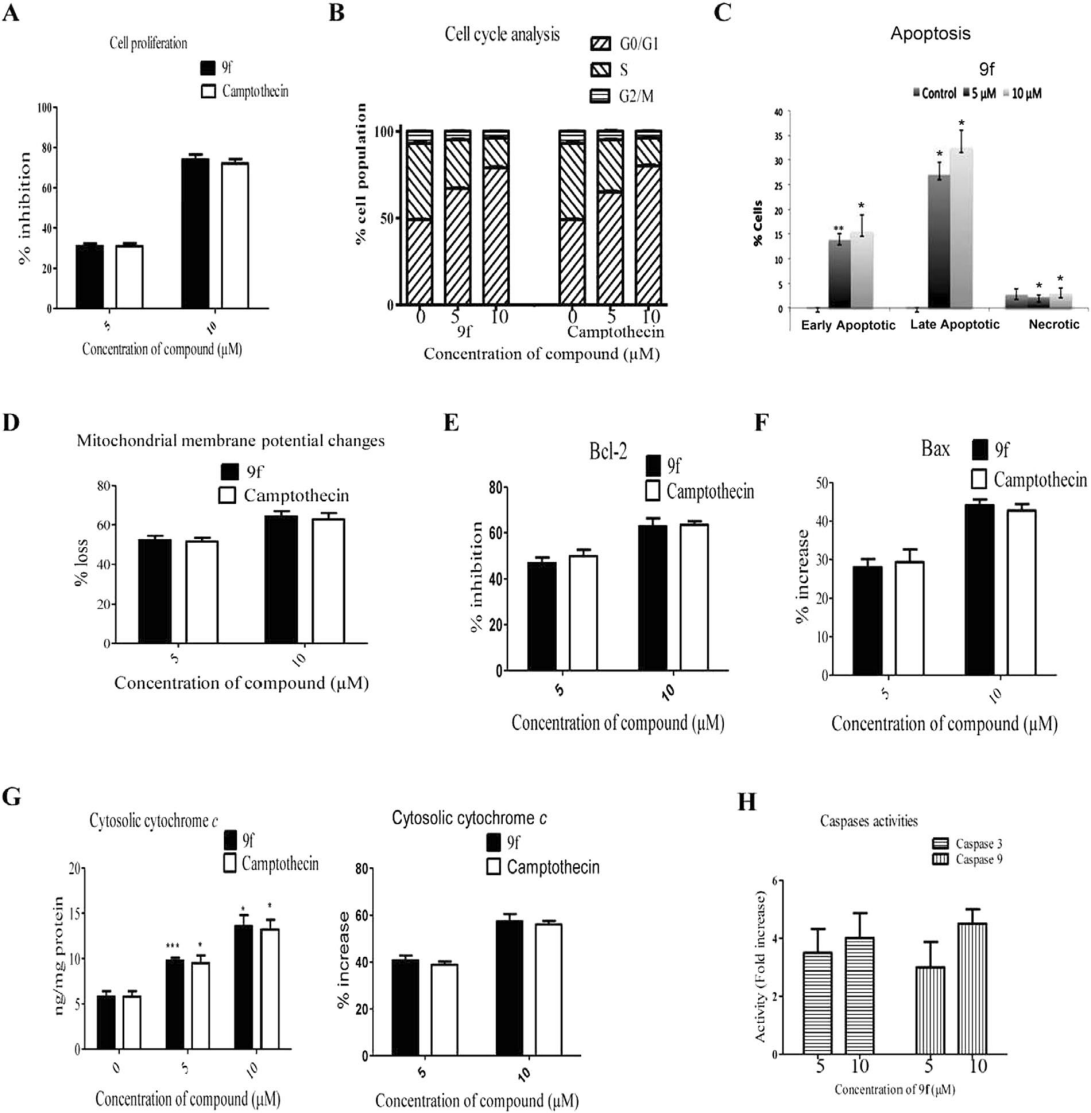
Pro-apoptotic potential of 9f in HL-60 cells. Panel A: Antiproliferative effect of **9f** in HL-60 at 24 h. Panel B: Effect of **9f** on cell cycle distribution of HL-60 cells. Panel C: Apoptotic cell population in **9f**-treated HL-60 cells. Panel D: Effect of **9f** on mitochondrial membrane potential in HL-60 cells. Panel E: Effect of **9f** on Bcl-2 levels represented in percent inhibition in treated and untreated HL-60 cells. Panel F: Distribution of Bax levels represented in percent increase in **9f** treated HL-60 cells. Panel G: Effect of **9f** in cytosolic cytochrome *c* levels represented in ng/ml and percent increase in treated and untreated HL-60 cells. Panel H: Effect of **9f** on caspases 3 and 9 activities represented in fold increase in treated HL-60 cells as compared to untreated. Each bar represents mean ± S.E.M. of three independent experiments; *P < 0.05, **P < 0.01 *versus* untreated control.

Pro-apoptotic potential of **9f** was also evaluated in HL-60 cells. Panel A of Fig. 6 represents the anti-proliferative effect of **9f** against HL-60 cells at 24 h. **9f** exhibited better antiproliferative effect than camptothecin (positive control) against HL-60 cells. In cell cycle analysis, dose-responsive accumulation of HL-60 cells in the G0/G1 phase of cell cycle was observed upon treatment with **9f** for 24 h (Panel B of Fig. 6). This demonstrates the cell cycle arrest at G0/G1 phase (Supplementary Figure 30). As shown in panel C of Fig. 6, apoptotic cell death of HL-60 cancer cells by **9f** was quantified by flow cytometry using Annexin V-PI staining technique. The results showed an increase in the early apoptotic to late apoptotic cells in a dose-dependent fashion in **9f**-treated HL-60 cells (Panel C of Fig. 6; Supplementary Figure 31).

**Figure 7:**
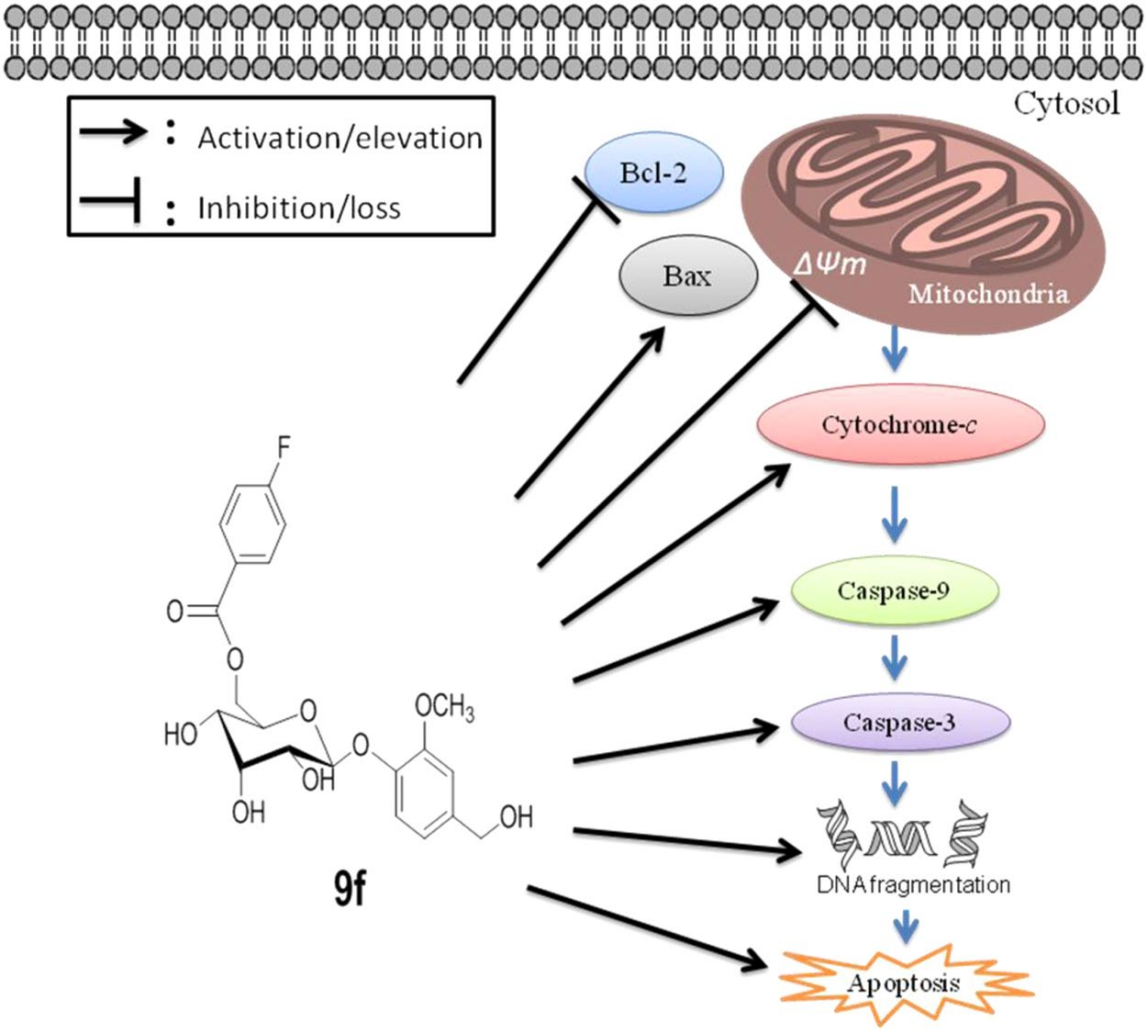
A diagram represents that 9f-induces apoptosis in MCF-7 breast and HL-60 cancer cells through mitochondria-mediated intrinsic pathway. 9f inhibits Bcl-2 levels and elevates Bax protein levels that may causes mitochondrial membrane potential (Δψm) loss and release of mitochondrial cytochrome *c* into cytosol, followed by activation of caspases 9 and 3 and DNA fragmentation.

**9f** caused 55.32 ± 2.14% and 65.56 ± 2.56% mitochondria membrane potential loss in HL-60 cells at 5 and 10 µM concentrations respectively compared to untreated cells (control) (Fig. 6 panel D). Compound **9f** showed dose dependent inhibition of Bcl-2 and increase in Bax protein levels in treated HL-60 cells as compared to untreated cells (Fig. 6, panel E and F). Overall, these results demonstrate the decreasing the ratio of Bcl-2/Bax in **9f**-treated HL-60 cancer cells (Fig. 6). This is an indication of apoptosis. Cytosolic cytochrome *c* levels are dose dependently increased in cytosolic fraction of **9f**-treated HL-60 cells (Fig. 6, panel G). Moreover, **9f** enhanced caspase-3 and -9 activities (Fig. 6, panel H), but it did not exhibit considerable effect on caspase-8 activity (data not showed) in HL-60 cells. These results confirmed that **9f** induces apoptosis in HL-60 cells via activation of mitochondria-mediated pathway (Fig. 7).

## Discussion

During last two decades, several phenolic glycoside (PG) esters have been isolated from plants and reported to possess anticancer activities^17–19^. 4-O-*β*-D-apifuranosyl-(1 → 2)-*β*-D-glucopyranosyl-2-hydroxy-6-methoxyacetophenone, a new phenolic glycoside was isolated from *Celosia argentea* and showed cytoxicity activity against SGC-7901 gastric and BEL-7404 hepatic cancer cells^20^. Ginnalins A–C, maple polyphenols, induced cell cycle arrest in colon and breast cancer cells by down regulating cyclins A and D1^21^. Salidroside, a phenolic glycoside from *Acer tegmentosum* Maxim, showed anticancer activity and induced apoptosis in human hepatocellular carcinoma Hep G2 cells^22^. Tao *et al*.^19^ isolated four new PG esters including saccharumoside B, from bark of the sugar maple tree and reported cytotoxic activity of saccharumoside B on human colon tumorigenic and nontumorigenic cell lines. However, the possible mechanism of anticancer natural product saccharumoside B in cancers is unknown. In present study, we have for first time synthesized saccharumoside B and its analogs (**9b**-**9f**, **10**) by Koenigs-Knorr reaction and evaluated anticancer activities of these compounds on various cancer cell lines including MCF-7 breast, MIA PaCa-2 pancreatic, DU145 prostate, CaCo-2 colon, HL-60 leukemia and HeLa cervical, and non-cancerous PBMC and MCF10A cells. Compounds (**9b**-**9f**, **10**) exhibited considerable cytotoxicity against cancer cells, but less cytotoxic against normal cells. Aforementioned results demonstrated that compounds are more cytotoxic towards cancerous cells than normal cells. Compound **9f** showed substantial anticancer activity against HL-60 and MCF-7 breast cancer cells among other compounds. **9f** showed better anticancer activity than saccharumoside B and comparable anticancer potency with camptothecin (positive control). Moreover, **9f** appeared less toxic to normal cells than to cancer cells *in vitro*.

Further, detailed studies of **9f** were carried out using MCF-7 and HL-60 cell line to establish the possible mechanism of action of anticancer compound **9f**. Cell cycle analysis results demonstrated that **9f** arrested MCF-7 and HL-60 cells at G0/G1. **9f** caused a dose-dependent increase in the early apoptotic to late apoptotic population in MCF-7 and HL-60 cells. These observations suggest that **9f** arrested cell cycle at G0/G1 phase and induced apoptosis. Apoptosis is the process of programmed cell death that may occurs through extrinsic and intrinsic pathways^23^. In intrinsic pathway, anti-apoptotic Bcl-2 family proteins inhibit mitochondrial outer membrane permeabilization. Pro-apoptotic Bax increases the permeability of mitochondrial outer membrane that leads to mitochondrial membrane potential loss and form mitochondrial apoptosis-induced channels. Followed by cytochrome *c* and other pro-apoptotic proteins are released into cytosol through the mitochondrial membrane pores. The released cytosolic cytochrome *c* activates caspase-9, which in turn activates caspases cascade including caspase-3 that leads to activation of ultimate biochemical events in apoptosis^23–25^. Hence, pro-apoptotic studies were carried out and results of these studies demonstrated that **9f** inhibited anti-apoptotic Bcl-2 and elevated pro-apoptotic Bax protein levels that caused mitochondrial membrane potential loss in MCF-7 and HL-60 cells. Subsequently, mitochondrial cytochrome *c* is released into cytosol and activated caspases 9 and 3. **9f** exhibited almost similar cellular effects in both MCF-7 and HL-60 cells, but it is more active against HL-60 cells. Overall, our study demonstrated that anticancer activity of saccharumoside B analog **9f** against HL-60 and MCF-7 cells by arresting the cell cycle at G0/G1 and inducting apoptosis via mitochondria-mediated intrinsic pathway (Fig. 7).

Chemotherapy is the most commonly used strategy for cancer treatment. However, side effects and dug resistance are the two major problems with available chemotherapeutic drugs^5–9^. Hence, discovery and development of new anticancer agents are highly desired for their clinical use. Previous studies demonstrated that new anticancer drugs with more potency have been developed from natural compounds through their structural modifications^26^. In this scenario, the current study demonstrates that **9f** (an analog of natural product saccharumoside-B) exhibited significant antiproliferative effect on cancer cells and is less toxic to normal cells *in vitro*. Our preliminary, but encouraging results, suggested **9f** as an excellent scaffold for further study in the field of cancer chemotherapy and needed *in vivo* anticancer and toxicity studies to develop it as an anticancer agent.

In conclusion, we have synthesized saccharumoside-B and their analogs (**9b**-**9n**, **10**) with good yields by the Koenigs-Knorr reaction. In antiproliferative studies, all synthesized compounds (**9b**-**9n**, **10**) showed considerable cytotoxic effect against MCF-7 breast, MIA PaCa-2 pancreatic, DU145 prostate, CaCo-2 colon, HL-60 leukemia and HeLa cervical cancer cell lines, but they showed less cytotoxicity against non-cancerous MCF10A and PBMC cells. The results demonstrated that compounds are more cytotoxic towards cancerous cells than normal cells. Among all tested compounds, **9f** showed significant anticancer activity in MCF-7 breast cancer and HL-60 leukemia cells. Moreover, cell cycle analysis in the MCF-7 and HL-60 cells has shown a significant increase in the G0/G1 and apoptotic population upon **9f** treatment, which is suggestive of induction of cell cycle arrest and apoptosis. Further, mitochondrial membrane potential loss, elevation of cytosolic cytochrome *c* and Bax levels, decrease in Bcl-2 levels, enhanced caspase-9 and -3 activities confirm the apoptosis-inducing potential of compound **9f** in MCF-7 breast cancer and HL-60 leukemia cell line via mitochondria-mediated intrinsic pathway. Thus, it is expected that **9f** can be developed as therapeutic drug for cancer based on future studies.

## Materials and Methods

### General information

Melting points were recorded on a Mel-Temp melting point apparatus and are uncorrected. Unless otherwise stated, all the materials were obtained from the commercial suppliers and are used without further purification. Chromatography was carried on silica gel (100–200 mesh). All the reactions were monitored by thin-layer chromatography and the spots were visualized under UV light. The ^1^H and ^13^C NMR spectra were recorded on Bruker FT-NMR spectrometer operating at 400 MHz for ^1^H and 100 MHz for ^13^C using TMS as an internal standard. Chemical shifts are expressed in δ (ppm) and coupling constants *J* in Hertz (Hz). Mass spectra were recorded on an Agilent 1100 LC/MSD.

### Synthesis of 4-(benzyloxy)-3-methoxybenzoyl chloride (7)

To a solution of vanillin (2.0 g, 13.15 mmol) and K_2_CO_3_ (5.44 g, 39.47 mmol) in acetone, benzyl chloride (2.3 g, 197.3 mmol) was added drop wise at room temperature. The reaction mixture was then stirred at room temperature for 4 h. After completion of the reaction (as indicated by TLC), the reaction mixture was filtered and concentrated under reduced pressure. The resultant residue was purified by silica gel column chromatography using hexane/ethyl acetate as eluents to afford 4-(benzyloxy)-3-methoxy benzaldehyde as off-white solid.

Aq. KOH (4.4 g, 20.2 mmol) was added to 4-(benzyloxy)-3-methoxy benzaldehyde (5.0 g, 20.06 mmol) at r.t. and the reaction mixture was heated at 180 °C for 1 h. After the completion of the reaction (as indicated by TLC), the reaction mixture was poured into crushed ice and acidified with conc. HCl up to a pH~2–3. The obtained solid was filtered, washed with distilled water and dried under reduced pressure. The obtained solid was dissolved in SOCl_2_ and allowed to stir at 100 °C for 2 h under anhydrous conditions. Excess of SOCl_2_ was removed under high vacuum and the obtained residue was used for the next step without further purification.

### Synthesis of pentaacetyl-α-D-glucose (2)

To a solution of α-D-glucose **1** (50.0 g, 280 mmol) in glacial acetic acid and acetic anhydride (162.8 mL) and perchloric acid in acetic anhydride (10 mL) was added drop wise using an addition funnel at 0 °C and allowed to stir at room temperature for 1 h. After completion of the reaction as monitored by TLC, the reaction mixture was poured into ice-cold water, and obtained solid was filtered, dried to get pure compound **2**.

### Synthesis of α-D-acetobromo glucose (3)

To a solution of pentaacetyl-α-D-glucose **2** (15 g, 38.4 mmol) in CH_2_Cl_2_, HBr in glacial acetic acid (23.9 g, 96.0 mmol) was added slowly at 0 °C. The reaction was then allowed to stir at room temperature for 1 h. After completion of the reaction as monitored by TLC, the reaction mixture was quenched using distilled water and extracted with CHCl_3_, the organic layer was dried by using anhydrous Na_2_SO_4_, and concentrated under vacuum to obtain compound **3**.

### General synthesis of tetra-acetyl phenolic glycosides (5a-n)

To a solution of **3** (2.0 g, 4.8 mmol) in CH_2_Cl_2_ and water (1:1), K_2_CO_3_ (0.88 g, 5.82 mmol), catalytic amount of aliquat-336 and corresponding 4-hydroxy benzaldehyde **4**(**a-n**) were added and stirred at 50 °C for overnight. After completion of the reaction (as indicated by TLC), the reaction mixture was quenched with ice-cold water and extracted with ethyl acetate. The organic layer was washed with water, brine and evaporated under reduced pressure to get crude. The crude residue was purified by silica gel column chromatography to afford pure compounds **5**(**a-n**).

### General synthesis of phenolic glycosides (6a-n)

To a solution of a compound **5**(**a-n**) (0.7 g, 1.4 mmol) in MeOH (5 mL), NaOMe (0.16 g, 2.9 mmol) was added and stirred at room temperature for 1 h. After completion of the reaction as monitored by TLC, methanol was evaporated and the residue was partitioned between water and ethyl acetate. The organic layer was evaporated, obtained crude was purified by silica gel column chromatography to get pure compound **6**(**a-n**).

### General synthesis of esters of 4-O-(β-D-glucopyranosyl) phenolic aldehydes (8a-n)

To a solution of compound **6**(**a-n**) (0.45 g, 1.4 mmol) in DCM, pyridine (14.8 mmol) and corresponding benzoyl chloride **7**(**a-n**) (0.59 g, 2.1 mmol) in CH_2_Cl_2_ were added at 0 °C. After completion of the reaction as monitored by TLC, the reaction mixture was quenched with ice-cold water, extracted with ethyl acetate. The organic layer was washed with water, brine solution. The residue was purified by silica gel column chromatography to afford pure compound **8**(**a-n**).

### General synthesis of glycosyl esters (9a-n)

To a solution of compound **8**(**a-n**) (0.2 g, 0.36 mmol) in MeOH, NaBH_4_ (0.02 g, 0.54 mmol) was added portionwise at 0 °C and the reaction was allowed to stir at room temperature for 30 min. After the completion of the reaction as monitored by TLC, methanol was evaporated and the residue was quenched with ice-cold water, acidified with 2N HCl (pH~2–3) and extracted with chloroform. The organic layer washed with water, brine and dried over anhy.Na_2_SO_4_ and evaporated under reduced pressure. The obtained residue was purified by using column chromatography to afford pure compound **9**(**a-n**) (Fig. 1).

### Synthesis of saccharumoside-B (10)

To a solution of compound-**9a** (150 mg, 2.7 mmol) in MeOH, TEA (0.07 mL, 0.54 mmol) and 5% Pd/CaCO_3_ were added and the reaction mixture was stirred at room temperature under H_2_ atmosphere (Fig. 2). After completion of the reaction, as determined by TLC, the reaction mass was filtered through a celite bed and the filtrate was evaporated under reduced pressure to get crude compound. The crude was purified by using column chromatography to afford pure compound **10**.

### Characterization data of compounds

#### (6-(4-(hydroxymethyl)-2-methoxyphenoxy)-tetrahydro-3,4,5-trihydroxy-2H-pyran-2-yl) methyl 4-hydroxy-3-methoxy benzoate (**10**)

Off- white solid, Mp: 200–202 °C; [α]^22.3^ -31.8 (*c* 0.5, MeOH). ^1^H NMR (400 MHz, MeOH-*d*_4_): *δ* 7.46 (dd, *J* = 1.6, 8.4 Hz, 1H), 7.42 (d, *J* = 2.0 Hz, 1H,), 6.92 (d, *J* = 8.4 Hz, 1H), 6.88 (d, *J* = 2.0 Hz, 1H), 6.76 (d, *J* = 8.4 Hz, 1H), 6.53 (d, *J* = 7.2 Hz, 1H), 4.78 (d, *J* = 7.2 Hz, 1H), 4.56 (dd, *J* = 1.6, 11.6 Hz, 1H), 4.39 (s, 2H), 4.28 (dd, *J* = 7.2, 12.0 Hz, 1H), 3.75 (s, 3H), 3.74 (s, 3H), 3.64 (ddd, *J* = 1.6, 7.2, 11.6 Hz, 1H), 3.44–3.40 (m, 2H), 3.34 (d, *J* = 9.6 Hz, 1H). ^13^C NMR (100 MHz, MeOH-*d*_4_): δ 167.9, 153.1, 150.9, 148.8, 147.1, 137.9, 125.3, 122.6, 120.6, 118.2, 116.0, 112.8, 102.9, 77.9, 75.7, 74.9, 72.1, 65.1, 64.9, 56.8, 56.6. LC-MS (ESI, positive ion mode): *m*/*z* 489.3 [M + Na]^+^. Anal. Calcd for C_22_H_26_O_11_: C, 56.65; H, 5.62; Found: C, 56.60; H, 5.66.

#### (6-(4-(hydroxymethyl)-2-methoxyphenoxy)-tetrahydro-3,4,5-trihydroxy-2H-pyran-2-yl)methyl benzoate (**9b**)

Off- white solid, Mp: 187–189 °C; [α]^22.0^ -31.88 (*c* 0.5, MeOH). ^1^H NMR (400 MHz, DMSO-*d*_6_): δ 8.00 (d, *J* = 7.2 Hz, 2H), 7.74 (t, *J* = 7.6 Hz,1H), 7.61 (t, *J* = 8.0 Hz, 2H), 7.09 (d, *J* = 8.4 Hz, 1H), 6.98 (s,1H), 6.72 (d, *J* = 8.4 Hz, 1H), 4.66 (d, *J* = 11.2 Hz, 1H), 4.46 (d, *J* = 5.6 Hz, 2H), 4.30 (dd, *J* = 4.0, 7.6 Hz, 1H), 3.38 (s, 3H). ^13^C NMR (100 MHz, DMSO-*d*_6_): δ 165.5, 148.7, 144.9, 136.5, 133.3, 129.7, 128.7, 118.3, 115.2, 111.1, 99.9, 76.6, 73.7, 73.2, 70.1, 64.3, 62.6, 55.6. LC-MS (ESI, positive ion mode): *m*/*z* 443.3 [M + Na]^+^. Anal. Calcd for C_21_H_24_O_9_: C, 59.99; H, 5.75. Found: C, 59.92; H, 5.80.

#### (6-(4-(hydroxymethyl)-2-methoxyphenoxy)-tetrahydro-3,4,5-trihydroxy-2H-pyran-2-yl)methyl 4-methylbenzoate (**9c**)

Off- white solid, Mp: 228–230 °C; [α]^21.1^ -31.8 (*c* 0.5, MeOH). ^1^H NMR (400 MHz, DMSO-*d*_6_): δ 7.89 (d, *J* = 7.6 Hz, 2H), 7.40 (d, *J* = 8.0 Hz, 1H), 7.07 (d, *J* = 8.0 Hz, 1H), 6.98 (s, 1H), 6.71 (d, *J* = 8.0 Hz, 1H), 4.99 (d, *J* = 6.4 Hz, 1H), 4.64 (d, *J* = 11.2 Hz, 1H), 4.46 (d, *J* = 5.6 Hz, 2H), 4.27 (dd, *J* = 7.6, 11.6 Hz,1H), 3.79 (s, 3H), 3.76- 3.29 (m, 4H), 2.46 (s, 3H). ^13^C NMR (100 MHz, DMSO-*d*_6_): δ 165.5, 148.8, 145.1, 143.6, 136.5, 129.2, 129.1, 127.0, 118.3, 115.3, 111.6, 100.1, 76.7, 73.8, 73.2, 70.2, 64.1, 62.7, 55.7, 21.2. LC-MS (ESI, positive ion mode): *m*/*z* 457.3 [M + Na]^+^. Anal. Calcd for C_22_H_26_O_9_: C, 60.82; H, 6.03; Found: C, 60.78; H, 6.10.

#### (6-(4-(hydroxymethyl)-2-methoxyphenoxy)-tetrahydro-3,4,5-trihydroxy-2H-pyran-2-yl)methyl 3,5-dimethoxybenzoate (**9d**)

Off- white solid, Mp: 199–201 °C; [α]^20.7^ -32.7 (*c* 0.5, MeOH).^1^H NMR (400 MHz, DMSO-*d*_6_): δ 7.17 (s, 2H), 7.07 (d, *J* = 8.8 Hz, 1H), 7.00 (s, 1H), 6.78 (s, 1H), 6.71 (d, *J* = 8.0 Hz, 1H), 4.71 (d, *J* = 11.6 Hz, 1H), 4.57 (brs, 1H), 4.51 (brs, 2H), 4.43 (dd, *J* = 7.2, 8.4, 1H), 3.83 (s, 6H), 3.79 (s, 3H), 3.75 (brs, 1H), 3.58–3.44 (m, 3H). ^13^C NMR (100 MHz, DMSO-*d*_6_): δ 167.5, 162.4, 157.9, 150.8, 147.1, 147.0, 137.8, 133.2, 120.8, 120.6, 118.3, 117.9, 112.7, 108.9, 77.8, 77.7, 75.6, 74.9, 72.0, 71.9, 56.7, 56.1. LC-MS (ESI, positive ion mode): *m*/*z* 503.3 [M + Na]^+^, 519.3 [M + K]^+^. Anal. Calcd. for C_23_H_28_O_11_: C, 57.50; H, 5.87; Found: C, 57.45; H, 5.90.

#### (6-(4-(hydroxymethyl)-2-methoxyphenoxy)-tetrahydro-3,4,5-trihydroxy-2H-pyran-2-yl)methyl 3,4,5-trimethoxybenzoate (**9e**)

Off- white solid, Mp: 168–170 °C; [α]^21.7^ -33.7 (*c* 0.5, MeOH). ^1^H NMR (400MHz, DMSO-*d*_6_): δ 7.47 (d, *J* = 10.0 Hz, 1H), 7.23 (s, 1H), 6.98 (d, *J* = 8.0 Hz, 1H), 6.77 (dd, *J* = 2.0, 6.4 Hz, 1H), 4.66–3.8 (m, 7H), 3.79 (s, 12H). ^13^C-NMR (100 MHz, DMSO-*d*_6_): δ 154.6, 149.2, 146.7, 138.5, 120.5, 118.7, 112.8, 109.0, 108.9, 108.4, 107.9, 103.3, 76.1, 73.4, 71.9, 66.9, 64.9, 60.6, 56.9. LC-MS (ESI, positive ion mode): *m*/*z* 533.1 [M + Na]^+^. Anal. Calcd for C_24_H_30_O_12_: C, 56.47; H, 5.92. Found: C, 56.43; H, 5.96.

#### (6-(4-(hydroxymethyl)-2-methoxyphenoxy)-tetrahydro-3,4,5-trihydroxy-2H-pyran-2-yl)methyl 4-fluorobenzoate (**9f**)

Off- white solid, Mp: 209–211 °C; [α]^21.8^ -30.56 (*c* 0.5, MeOH). ^1^H NMR (400 MHz, DMSO-*d*_6_): δ 7.98 (t, *J* = 6.8 Hz, 2H), 7.37 (t, *J* = 8.0 Hz, 2H), 7.01 (d, *J* = 7.6 Hz, 1H), 6.91 (s, 1H), 6.66 (d, *J* = 8.0 Hz, 1H), 5.32 (brs, 1H), 5.09 (d, *J* = 4 Hz, 1H), 4.95 (d, *J* = 6 Hz, 1H), 4.58 (d, *J* = 11.6 Hz, 1H), 4.39 (s, 2H), 4.23 (brs, 1H). ^13^C NMR (100 MHz, DMSO-*d*_6_): δ 153.6, 137.6, 133.8, 131.6, 131.0, 129.9, 129.4, 114.9, 102.8, 77.6, 75.2, 73.6, 70.6, 66.4, 62.9. LC-MS (ESI, positive ion mode): *m*/*z* 461.3 [M + Na]^+^. Anal. Calcd for C_21_H_23_FO_9_: C, 57.53; H, 4.33. Found: C, 57.48; H, 4.38.

#### (6-(4-(hydroxymethyl)-2-methoxyphenoxy)-tetrahydro-3,4,5-trihydroxy-2H-pyran-2-yl)methyl 2-iodobenzoate (**9g**)

Off- white solid, Mp: 128–130 °C [α]^21.3^ -31.8 (*c* 0.5, MeOH). ^1^H NMR (400 MHz, MeOH-*d*_4_): δ 7.77 (dd, *J* = 1.2, 7.6 Hz, 1H), 7.72 (dd, *J* = 1.6, 7.6 Hz, 1H), 7.25 (dd, *J* = 2.0, 8.0 Hz, 1H), 7.21 (d, *J* = 3.6, 4.0 Hz, 1H), 7.09 (d, *J* = 8.0 Hz, 1H), 7.01 (d, *J* = 2.0 Hz,1H), 4.93 (d, *J* = 7.2 Hz, 1H), 4.70 (dd, *J* = 2.0, 12.0 Hz, 1H), 4.52 (s, 2H), 4.44 (dd, *J* = 5.2, 7.2 Hz, 1H), 3.86 (s, 3H), 3.78 (ddd, *J* = 2.0, 7.6, 9.2 Hz, 3H), 3.57–3.44 (m, 3H). ^13^C NMR (100 MHz, MeOH-*d*_4_): δ 168.0, 151.2, 146.9, 142.1, 138.1, 136.8, 133.7, 131.2, 129.2, 120.6, 118.6, 112.7, 102.8, 77.8, 75.5, 74.9, 71.9, 65.9, 64.9, 56.7. LC-MS (ESI, positive ion mode): *m*/*z* 569.2 [M + Na]^+^. Anal. Calcd for C_21_H_23_IO_9_: C, 46.17; H, 4.24; Found: C, 46.12; H, 4.30.

#### (6-(4-(hydroxymethyl)-2-methoxyphenoxy)-tetrahydro-3,4,5-trihydroxy-2H-pyran-2-yl)methyl nicotinate (**9h**)

Off- white solid, Mp: 194–196 °C; [α]^22.3^ -27.18 (*c* 0.5, MeOH). ^1^H NMR (400 MHz, DMSO-*d*_6_): δ 9.07 (s, 1H), 8.78 (d, *J* = 7.6 Hz, 1H), 8.39 (dd, *J* = 1.6, 5.6 Hz, 1H), 7.59 (dd, *J* = 2.8, 8.0 Hz, 1H), 7.06 (d, *J* = 8.4 Hz, 1H,), 7.01 (s, 1H), 6.70 (d, *J* = 8.4 Hz, 1H), 4.94 (d, *J* = 7.2 Hz, 1H), 4.76 (dd, *J* = 2.8, 12.4 Hz, 1H), 4.49 (brs, 2H), 4.47 (d, *J* = 4.4 Hz, 1H), 3.86 (s, 3H), 3.80–3.48 (m, 5H). ^13^C NMR (100 MHz, DMSO-*d*_6_): δ 166.0, 154.1, 151.2, 151.0, 146.8, 139.0, 138.0, 127.9, 125.3, 120.4, 118.2, 112.7, 102.6, 77.8, 75.4, 74.9, 72.0, 65.8, 64.9, 56.7. LC-MS (ESI, positive ion mode): *m*/*z* 422 [M + H]^+^, 444.3 [M + Na]^+^. Anal. Calcd for C_20_H_23_NO_9_: C, 57.00; H, 5.50. Found: C, 56.93; H, 5.56.

#### (6-(4-(hydroxymethyl)-2-methoxyphenoxy)-tetrahydro-3,4,5-trihydroxy-2H-pyran-2-yl)methyl 3,5-dinitrobenzoate (**9i**)

Off- white solid, Mp: 186–188 °C; [α]^21.3^ -29.16 (*c* 0.5, MeOH). ^1^H NMR (400 MHz, DMSO-*d*_6_): δ 8.95 (d, *J* = 6.4 Hz, 2H), 6.90 (d, *J* = 8.4 Hz, 2H), 6.80 (s, 1H), 6.53 (t, *J* = 8.0 Hz, 1H), 4.77 (bros, 1H), 4.43 (s, 2H), 3.74 (s, 3H), 3.65- 3.36 (m, 6H). ^13^C NMR (100MHz, DMSO-*d*_6_): δ 169.5, 133.7, 132.4, 130.1, 129.9, 125.6, 123.5, 120.8, 118.7, 112.9, 103.2, 79.5, 77.9, 74.9, 71.5, 69.2, 65.0, 62.6, 56.8. LC-MS (ESI, negative ion mode): *m*/*z* 547.2 [M + 2H_2_O-H]^−^. Anal. Calcd for C_21_H_22_N_2_O_13_: C, 49.42; H, 4.34; Found: C, 49.40; H, 4.38.

#### (6-(4-(hydroxymethyl)-2,6-dimethoxyphenoxy)-tetrahydro-3,4,5-trihydroxy-2H-pyran-2-yl)methyl benzoate (**9j**)

Off- white solid, Mp: 180–182 °C; [α]^21.2^ -26.4 (*c* 0.5, MeOH). ^1^H NMR (400 MHz, DMSO-*d*_6_): δ 7.80 (d, *J* = 7.2 Hz, 2H); 7.65 (t, *J* = 7.6 Hz, 1H), 7.52 (t, *J* = 7.6 Hz, 2H), 6.59 (s, 1H), 4.87 (d, *J* = 5.6 Hz, 1H), 4.50 (d, *J* = 10.8 Hz, 1H), 4.41 (d, *J* = 5.6 Hz, 2H), 4.19 (dd, *J* = 6.8, 11.6 Hz, 1H), 3.66 (s, 6H), 3.27 (brs, 3H). ^13^C NMR (100 MHz, DMSO-*d*_6_): δ 165.5, 152.6, 138.5, 133.2, 132.7, 129.7, 129.0, 128.6, 104.2, 102.6, 76.3, 74.1, 73.8, 70.2, 64.0, 62.9, 56.1. LC-MS (ESI, positive ion mode): *m*/*z* 473.2 [M + Na]^+^, 489.2 [M + K]^+^. Anal. Calcd for C_22_H_26_O_10_: C, 58.66; H, 5.82. Found: C, 58.62; H, 5.87.

#### (6-(4-(hydroxymethyl)-2,6-dimethoxyphenoxy)-tetrahydro-3,4,5-trihydroxy-2H-pyran-2-yl)methyl 4-methylbenzoate (**9k**)

Off- white solid, Mp: 201–203 °C; [α]^22.3^ -27.16 (*c* 0.5, MeOH). ^1^H NMR (400 MHz, MeOH-*d*_4_): δ 7.69 (d, *J* = 8 Hz, 2H), 7.31 (d, *J* = 8 Hz, 2H), 6.59 (s, 2H), 4.86 (d, *J* = 6.4 Hz, 1H), 4.49 (d, *J* = 11.6 Hz, 1H) 4.41 (s, 2H), 4.17 (ddd, *J* = 2.8, 6.4, 11.6 Hz, 1H), 3.67 (s, 6H), 2.39 (s, 3H). ^13^C NMR (100 MHz, MeOH-*d*_4_): δ 165.5, 152.6, 143.5, 138.4, 129.2, 129.1, 127.0, 104.3, 102.7, 76.4, 74.1, 73.9, 70.2, 63.9, 62.9, 56.1, 21.1. LC-MS (ESI, positive ion mode): *m*/*z* 487.3 [M + Na]^+^, 503.2 [M + K]^+^. Anal. Calcd for C_23_H_28_IO_10_: C, 59.48; H, 6.08. Found: C, 59.42; H, 6.14.

#### (6-(4-(hydroxymethyl)-2,6-dimethoxyphenoxy)-tetrahydro-3,4,5-trihydroxy -2H-pyran-2-yl)methyl 3,5-dimethoxybenzoate (**9l**)

Off- white solid, Mp: 131–133 °C; [α]^22.4^ -27.16 (*c* 0.5, MeOH). ^1^H NMR (400 MHz, DMSO-*d*_6_): δ 7.08 (dd, *J* = 2.8, 8.8 Hz, 1H), 6.97 (d, *J* = 2.0 Hz, 2H), 6.79 (s, 1H), 6.56 (s, 1H), 4.85 (d, *J* = 5.6 Hz, 1H), 4.47 (d, *J* = 6.8 Hz, 1H), 4.44 (s, 2H), 3.78 (s, 6H), 3.67 (s, 6H), 3.39–3.26 (m, 5H). ^13^C NMR (100 MHz, DMSO-*d*_6_): δ 165.1, 160.3, 152.5, 138.4, 132.8, 131.7, 106.9, 106.8, 104.8, 104.2, 102.7, 76.2, 74.1, 73.8, 70.0, 64.1, 62.9, 56.1, 55.5, 55.4. LC-MS (ESI, positive ion mode): *m*/*z* 533.3 [M + Na]^+^. Anal. Calcd for C_24_H_30_O_12_: C, 56.47; H, 5.92. Found: C, 56.40; H, 5.97.

#### (6-(4-(hydroxymethyl)-2,6-dimethoxyphenoxy)-tetrahydro-3,4,5-tri hydroxy -2H-pyran-2-yl)methyl 4-fluorobenzoate (**9m**)

Off- white solid, Mp: 170–172 °C; [α]^22.0^ -18.68 (*c* 0.5, MeOH). ^1^H NMR (400 MHz, DMSO-*d*_6_): δ 7.89 (brs 1H), 7.39 (t, *J* = 8.8 Hz, 2H), 6.64 (s, 2H), 4.93 (brs, 1H), 4.55 (d, *J* = 11.6 Hz, 1H), 4.46 (brs, 2H), 4.24 (dd, *J* = 5.6, 9.2 Hz, 1H), 3.72 (m, 4H), 3.37 (m, 6H). ^13^C NMR (100 MHz, DMSO-*d*_6_): δ 152.6, 138.4, 132.6, 132.1, 132.0, 131.9, 131.8, 115.8, 102.5, S76.3, 74.1, 73.8, 70.3, 64.2, 62.9, 56.1. LC-MS (ESI, positive ion mode): *m*/*z* 491.2 [M + Na]^+^, 507.2 [M + K]^+^. Anal. Calcd for C_22_H_25_FO_10_: C, 56.41; H, 5.38. Found: C, 56.37; H, 5.40.

#### (6-(4-(hydroxymethyl)-2,6-dimethoxyphenoxy)-tetrahydro-3,4,5-tri hydroxy-2H-pyran-2-yl)methyl 2-iodobenzoate (**9n**)

Off- white solid, Mp: 124–126 °C; [α]^21.6^ -0.92 (*c* 0.5, MeOH). ^1^H NMR (400 MHz, DMSO-*d*_6_): δ 7.98 (d, *J* = 8.0 Hz, 1H), 7.46 (dt, *J* = 2.0, 7.6 Hz, 1H), 7.42 (dd, *J* = 1.6, 7.6 Hz, 1H), 7.27 (ddd, *J* = 0.8, 1.2, 1.8 Hz, 1H), 6.59 (s, 2H), 4.88 (d, *J* = 6.4 Hz, 1H), 4.46 (d, *J* = 11.6 Hz, 1H), 4.41 (d, *J* = 5.6 Hz, 2H), 4.25 (dd, *J* = 6.8, 6.8 Hz, 1H), 3.67 (s, 6H), 3.27–3.25 (m, 3H). ^13^C NMR (100 MHz, DMSO-*d*_6_): δ 166.0, 152.6, 140.6, 138.5, 135.4, 132.9, 132.6, 130.3, 128.1, 104.1, 102.6, 94.1, 76.2, 74.0, 73.7, 70.1, 64.7, 62.9, 56.1. LC-MS (ESI, positive ion mode): *m*/*z* 599.1 [M + Na]^+^, 615.0 [M + K]^+^. Anal. Calcd for C_22_H_25_IO_10_: C, 45.85; H, 4.37. Found: C, 45.81; H, 4.40.

### Chemicals and reagents

RPMI-1640, minimal essential medium (MEM), Dulbecco’s modified eagle medium (DMEM), DNase-free RNase, 3-(4,5-dimethylthiazole-2-yl)-2,5-diphenyltetrazolium bromide (MTT), camptothecin were purchased from Sigma Chemical Company (St. Louis, MO, USA). Other reagents used were of analytical grade and available locally.

### Cell lines

Colon CaCo-2 pancreatic MIA PaCa-2 and promyelocytic human leukemia HL-60 cell lines were procured from European Collection of Cell Cultures (ECACC, Salisbury, Wiltshire, UK). Breast cancer cell line MCF-7 was obtained from National Cancer Institute (Frederick, MD, USA). Human prostate carcinoma DU145, human mammary epithelial MCF-10A and human cervical cancer HeLa cell lines were obtained from American Type Culture Collection (Manassas, VA, USA). Human peripheral blood mononuclear cells (PBMC) were collected from whole blood by Ficoll Plague method^27^. Cells were grown in RPMI-1640/DMEM/MEM medium containing 10% fetal calf serum (FCS), 100 mg of streptomycin and 100 units penicillin per ml medium. Cells were incubated in CO_2_ incubator at 37 °C with 95% humidity and 5% CO_2_ gas environment. Cells were treated with tested compounds dissolved in DMSO, while the untreated control cultures received only the vehicle (DMSO, <0.2%).

### Cell proliferation by MTT assay

MTT assay was performed to assess the antiproliferative activities of compounds^27, 28^. Cells were treated with compounds at various concentrations for 24 h or 48 h. 20 µL of freshly prepared MTT reagent was added to cells and incubated at 37 °C for 2 h. The supernatant growth medium was removed and replaced with DMSO (100 µL) to dissolve the formazan crystals. The optical density (absorbance) was read at 570 nm with reference wavelength of 620 nm. Three individual experiments were performed and results were expressed as percent inhibitions at various doses of each compound. IC_50_ means the concentration of the compound, which inhibits 50% of cell growth^28^.

### Cell cycle analysis

Cancer cells (1 × 10^6^/ml) were treated with different concentrations of compound (5 and10 µM) for 24 h, fixed in cold 70% alcohol in PBS, washed, digested with DNase free RNase (400 µg/mL) at 37 °C for 45 min and stained with propidium iodide (10 µg/ml). Cells were analyzed for PI-DNA fluorescence by flow cytometerically using FACS CALIBUR (Becton Dickinson, Franklin Lakes, New Jersey, USA)^28–30^. The fluorescence intensity of subG0/GI cell fraction represents the dead cell population.

### Quantification of apoptosis

Apoptotic population of cancer cells was quantified by Annexin V-propidium iodide (PI) staining technique (Abcam, Cambridge, MA, USA) using a flow cytometer as per published technique^31^.

### Mitochondrial Membrane Potential Changes (*ΔΨm*) Measurement Assay

Mitochondrial membrane potential changes (*ΔΨm*) were determined by flow cytometry using rhodamine-123 (green-fluorescent dye)^32^. MIA PaCa-2 pancreatic cancer cells (1 × 10^6^ cells/well) were exposed to the isolated compound (5 µM) for 24 h. Further, rhodamine-123 (200 nM) was added to the cells, which were kept in dark for 35 min. The cells were further centrifuged and the pellet was washed with phosphate buffered saline (1 ml). The intensity of fluorescence in the cells represented the mitochondrial membrane potential change (*ΔΨm)*, which was measured using a flow cytometer^32^.

### Cytosolic cytochrome *c* estimation

Cells were collected by centrifugation at 600 *g* for 5 min at 4 °C. The cell pellets were washed once with ice-cold PBS and resuspended in cytosol extraction buffer (20 mM Hepes-KOH, pH 7.5, 10 mM KCl, 1.5 mM MgCl_2_, 1 mM sodium EDTA, 1 mM sodium EGTA, 1 mM dithiothreitol and 0.1 mM PMSF) containing 250 mM sucrose on ice for 10 min. The cells were homogenized with the grinder on ice. Further, the homogenates were centrifuged at 700 *g* for 10 min at 4 °C. The supernatants were centrifuged at 10,000 × g for 30 min at 4 °C. Supernatant was collected as cytosolic fraction.

Cytosolic cytochrome *c* levels were determined using quantitative sandwich enzyme immunoassay using microplate reader. Sample or standard (100 μL) was added into each well and incubated for 2 h at room temperature. The wells were washed several times with wash buffer. The plate was inverted and cleaned with paper toweling. Cytochrome *c* conjugate (200 μL) was added into each well and incubated for 2 h at room temperature. Wells were washed several times with wash buffer. Substrate solution (200 μL) was added into each well and incubated for 30 min at room temperature. Finally, stop solution (50 μL) was added into wells. The optical density (absorbance) of each well was determined using microplate reader at a 450 nm wavelength^32^.

### Caspases assays

Caspases-3, -8 and -9 assays were measured according to manufacturer’s instructions using a caspase colorimetric protease kit (Abcam, Cambridge, MA, USA). Cells were treated with compounds for 48 h. The cells were lysed by the addition of 50 μL of chilled cell lysis buffer and incubated on ice for 10 min. The resulting cell lysate was centrifuged for 1 min at 10,000 *g*, and the supernatant was collected. The cell lysate containing 75 mg of protein was incubated with 4 mL of 4 mmol/L pNA-conjugated substrates (DEVD-pNA, IETD-pNA and LEHD-pNA; substrates for caspase-3, -8 and -9, respectively) at 37 °C for 3 h. The amount of pNA released was measured at 405 nm using an ELISA microplate reader (Bio rad). The caspases activity was expressed as fold difference^32, 33^.

### Quantification of Bcl-2

The intracellular content of Bcl-2 was quantified using the human Bcl-2 ELISA kit (Abcam, Cambridge, MA, USA). For the sample preparation, the cells were lysed with 1X lysis buffer. After 1 h of incubation at room temperature, the sample was spun at 1,000 g for 15 min and the supernatant was used for Bcl-2 measurement. Briefly, 80 μL of sample diluent and 20 μL of sample were added into the wells coated with monoclonal antibody to human Bcl-2, and after that the wells were washed twice with the wash buffer. Next, 50 μL of biotin-conjugate were added into the wells. After 2 h of incubation at room temperature on a microplate shaker, 100 μL of streptavidin-horseradish peroxidase were added. The sample was incubated again for 1 h at room temperature on a microplate shaker. The solution was withdrawn and the wells were washed with 3X wash buffer. Immediately, 100 μL of TMB substrate solution were added. Finally, 100 μL of stop solution were added into each well to stop the enzyme reaction. The absorbance was read using an ELISA reader at 450 and 620 nm wavelengths. Bcl-2 protein concentration was determined from the standard protein graphic using the optical densities of the samples^32^.

### Quantification of Bax

Bax protein concentration determination was carried out using the Human Bax Enzyme Immunometric Assay kit (Assay Designs, Ann Arbor, MI, USA). The lysate (sample) preparation was carried out according to the manual. Following centrifugation (16,000 g for 15 min), the cells were resuspended in Modified Cell lysis buffer 4 [0.5 μL/mL of Sigma Protease Inhibitor Cocktail and 1 mM phenylmethylsulfonyl fluoride (PMSF)]. Cell lysate (100 μL) (sample) was added into the wells coated with monoclonal antibody to human Bax-α in triplicate. The plate was tapped gently to mix the contents. The sample was incubated at room temperature on a plate shaker for 1 h. The wells were emptied and washed with 5X wash buffer. After the final wash, the plate was tapped gently on a lint free paper towel to remove any remaining wash buffer. The sample was incubated again for 1 h at room temperature on a plate shaker after the addition of 100 μL of specific antibody (biotinylated monoclonal antibody to Bax-α) into each well. The wells were washed again with 5X wash buffer and emptied. Next, 100 μL of blue conjugate (streptavidin conjugated to horseradish peroxidase) was added into each well. The sample was left on a plate shaker for 30 min at room temperature. The wells were washed again as in the previous step. Solution (100 μL) of 3,3′,5,5′ tetramethylbenzidine (TMB) and H_2_O_2_ was added into each well. Finally, 100 μL of stop solution containing hydrochloric acid in water was added to stop the enzyme reaction. The optical densities were read at 450 and 570 nm using an ELISA reader. Bax protein concentration was determined from the standard protein graphic using the optical densities of the samples^32^.

### Statistical analysis

Data are presented as mean ± standard error of mean (S.E.M) from triplicate parallel experiments unless otherwise indicated. Statistical significance is performed using Student’s *t*-test. The difference of the statistical data of two groups: *p* ≤ 0.05 was considered as significant.

## Electronic supplementary material

Supplementary Figures
