# Publisher Correction: Synthesis of Saccharumoside-B analogue with potential of antiproliferative and pro-apoptotic activities

**DOI:** 10.1038/s41598-019-57392-w

**Published:** 2020-01-08

**Authors:** Srinuvasarao Rayavarapu, Nagendra Sastry Yarla, Sunanda Kumari Kadiri, Anupam Bishayee, Siddaiah Vidavalur, Ramu Tadikonda, Mahaboob Basha, Vijaya Rao Pidugu, Kaladhar S. V. G. K. Dowluru, Dhananjaya Bhadrapura Lakappa, Mohammad A. Kamal, Ghulam Md Ashraf, Vadim V. Tarasov, Vladimir N. Chubarev, Sergey G. Klochkov, George E. Barreto, Sergey O. Bachurin, Gjumrakch Aliev

**Affiliations:** 1 Department of Organic Chemistry, Foods, Drugs and Water, College of Science and Technology, Andhra University, Visakhapatnam, 530 003 Andhra Pradesh India; 2 Department of Biochemistry and Bioinformatics, School of Life Sciences, Institute of Science, GITAM University, Visakhapatnam, 530 045 Andhra Pradesh India; 3 Department of Animal Biology, University of Hyderabad, Hyderabad, 500 046 Telangana India; 4 Department of Microbiology, College of Science and Technology, Andhra University, Visakhapatnam, 530 003 Andhra Pradesh India; 5 Department of Pharmaceutical Sciences, College of Pharmacy, Larkin Health Sciences Institute, Miami, FL 33169 USA; 6 Excelra Knowledge Solutions Private Limited, NSL SEZ ARENA, IDA Uppal, Hyderabad, 500 039 Telangana India; 7 Department of Microbiology and Bioinformatics, Bilaspur University, Bilaspur, 495 001 Chhattisgarh India; 8 Toxinology/Toxicology and Drug Discovery Unit, Center for Emerging Technologies, Jain Global Campus, Jain University, Kanakapura Taluk, Ramanagara, 562 112 Karnataka India; 9 Enzymoics and Novel Global Community Educational Foundation, Hebersham, NSW Australia; 10 King Fahd Medical Research Center, King Abdulaziz University, Jeddah, Saudi Arabia; 11 Institute of Pharmacy and Translational Medicine, Sechenov First Moscow State Medical University, 119991 Moscow, Russia; 12 Institute of Physiologically Active Compounds of the Russian Academy of Sciences, Severniy Proezd, Chernogolovka, Moscow Region 1142432 Russia; 13 Departamento de Nutrición y Bioquímica, Facultad de Ciencias, Pontificia Universidad Javeriana, Bogotá, D. C. Colombia; 14 Instituto de Ciencias Biomédicas, Universidad Autónoma de Chile, Santiago, Chile; 15 “GALLY” International Biomedical Research Consulting LLC, San Antonio, TX 78229 USA; 16 School of Health Sciences and Healthcare Administration, University of Atlanta, Johns Creek, GA 30097 USA

Correction to: *Scientific Reports* 10.1038/s41598-017-05832-w, published online 16 August 2017

In the original version of this Article, Ghulam Md Ashraf was incorrectly affiliated with ‘Enzymoics and Novel Global Community Educational Foundation, Hebersham, NSW, Australia’. The correct affiliation is listed below.

King Fahd Medical Research Center, King Abdulaziz University, Jeddah, Saudi Arabia.

Additionally, within the Supplementary Information file originally published with this Article, authors Vijaya Rao Pidugu, Mohammad A. Kamal, Ghulam Md Ashraf, Vadim V. Tarasov, Vladimir N. Chubarev, Sergey G. Klochkov, George E. Barreto and Sergey O. Bachurin were omitted.

These errors have now been corrected in the PDF and HTML versions of the Article, and in the accompanying Supplementary material.

