# Supplementary material for: Synthesis of Saccharumoside-B analogue with potential of antiproliferative and pro-apoptotic activities
Source: Sci Rep. 2017 Aug 16;7:8309. doi: 10.1038/s41598-017-05832-w (PMC5559490; doi:10.1038/s41598-017-05832-w)
Supplement: Supplementary file 1 — Supplementary Figures [file 41598_2017_5832_MOESM1_ESM.doc]

Synthesis of Saccharumoside-B analogue with potential of antiproliferative and pro-apoptotic activities

Srinuvasarao Rayavarapu^1^, Nagendra Sastry Yarla^2,3^, Sunanda Kumari Kadiri^4^, Anupam

Bishayee^5^, Siddaiah Vidavalur^1^, Ramu Tadikonda^1^, Mahaboob Basha^1^, Vijaya Rao Pidugu^6^,

Kaladhar S. V. G. K. Dowluru^7^, Dhananjaya Bhadrapura Lakappa^8^, Mohammad A. Kamal^9,10^, Ghulam Md Ashraf^10^, Vadim V. Tarasov^11^, Vladimir N. Chubarev^11^, Sergey G. Klochkov^12^, George E. Barreto^13,14^, Sergey O. Bachurin^12^ & Gjumrakch Aliev^12,15,16^

^1^Department of Organic Chemistry, Foods, Drugs and Water, College of Science and Technology, Andhra University, Visakhapatnam, 530 003, Andhra Pradesh, India. ^2^Department of Biochemistry and Bioinformatics, School of Life Sciences, Institute of Science, GITAM University, Visakhapatnam, 530 045, Andhra Pradesh, India.

^3^Department of Animal Biology, University of Hyderabad, Hyderabad, 500 046, Telangana, India.

^4^Department of Microbiology, College of Science and Technology, Andhra University, Visakhapatnam, 530 003, Andhra Pradesh, India.

^5^Department of Pharmaceutical Sciences, College of Pharmacy, Larkin Health Sciences Institute, Miami, FL, 33169, USA.

^6^Excelra Knowledge Solutions Private Limited, NSL SEZ ARENA, IDA Uppal, Hyderabad, 500 039, Telangana, India.

^7^Department of Microbiology and Bioinformatics, Bilaspur University, Bilaspur, 495 001, Chhattisgarh, India.

^8^Toxinology/Toxicology and Drug Discovery Unit, Center for Emerging Technologies, Jain Global Campus, Jain University, Kanakapura Taluk, Ramanagara, 562 112, Karnataka, India. ^9^Enzymoics and Novel Global Community Educational Foundation, Hebersham, NSW, Australia.

^10^King Fahd Medical Research Center, King Abdulaziz University, Jeddah, Saudi Arabia. ^11^Institute of Pharmacy and Translational Medicine, Sechenov First Moscow State Medical University, 119991, Moscow, Russia.

^12^Institute of Physiologically Active Compounds of the Russian Academy of Sciences, Severniy Proezd, Chernogolovka, Moscow Region, 1142432, Russia.

^13^Departamento de Nutrición y Bioquímica, Facultad de Ciencias, Pontificia Universidad Javeriana, Bogotá, D. C., Colombia.

^14^Instituto de Ciencias Biomédicas, Universidad Autónoma de Chile, Santiago, Chile. ^15^“GALLY” International Biomedical Research Consulting LLC, San Antonio, TX, 78229, USA.

^16^School of Health Sciences and Healthcare Administration, University of Atlanta, Johns Creek, GA, 30097, USA.

Srinuvasarao Rayavarapu, Nagendra Sastry

Yarla and Sunanda Kumari Kadiri contributed equally to this work.

Correspondence and requests for materials should be addressed to G.A. (email: aliev03@gmail.com)

**Supplementary figures**

| 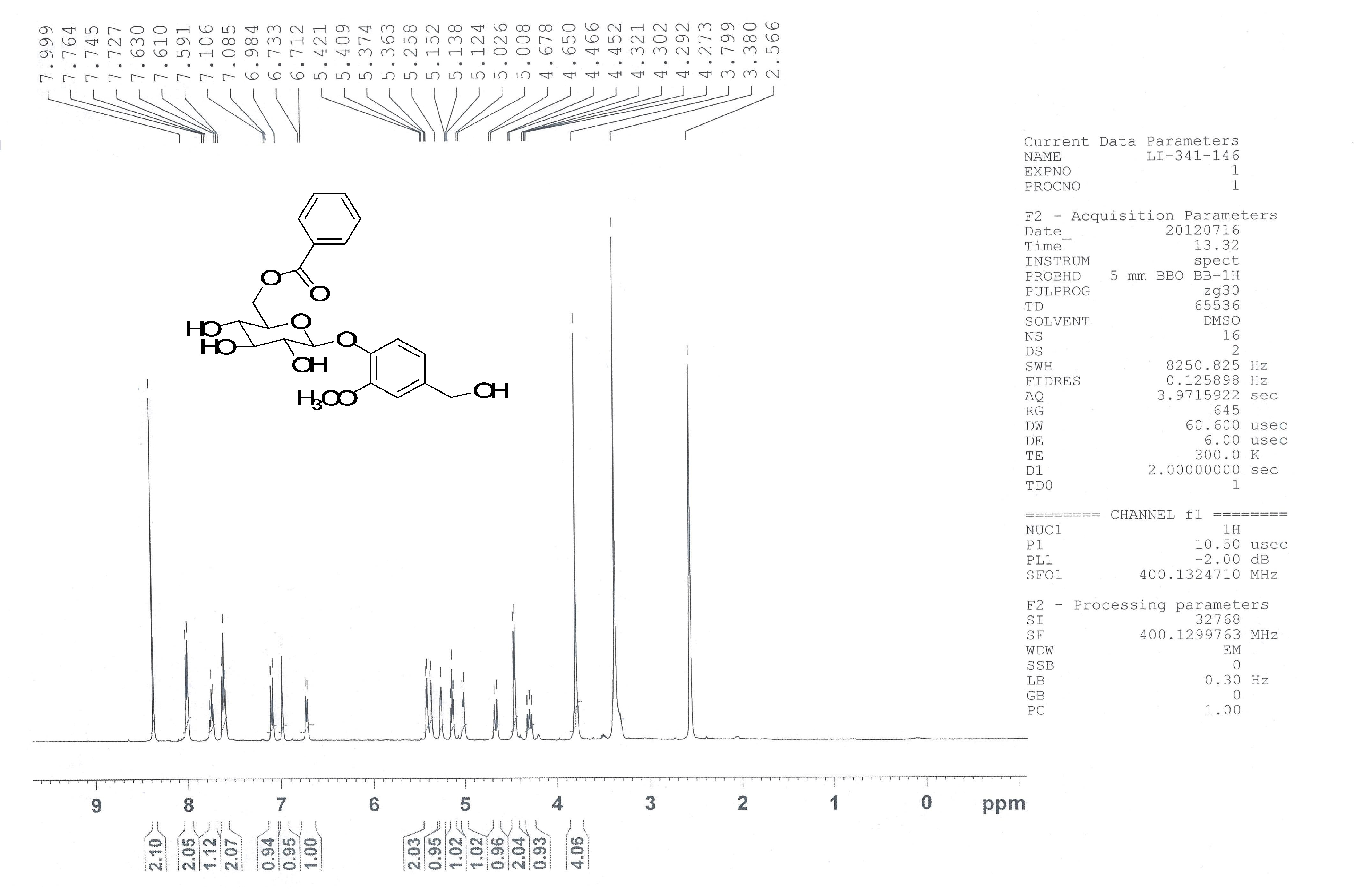 |
| --- |
|  |
| **Figure 1.** **^1^H NMR spectrum of compoumd-9b** |
| **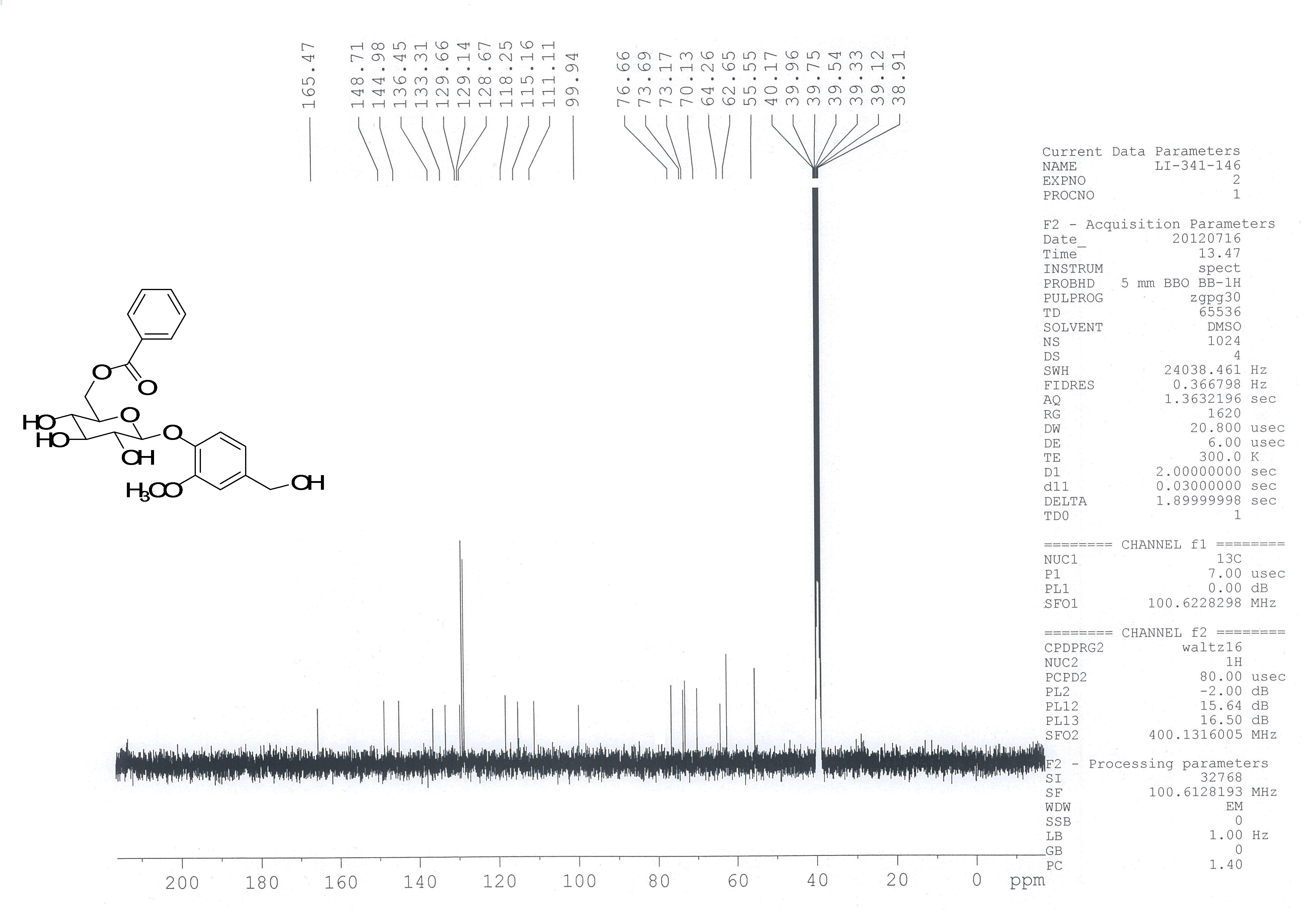** |
| **Figure 2. ^13^C NMR spectrum of compound-9b** |
| **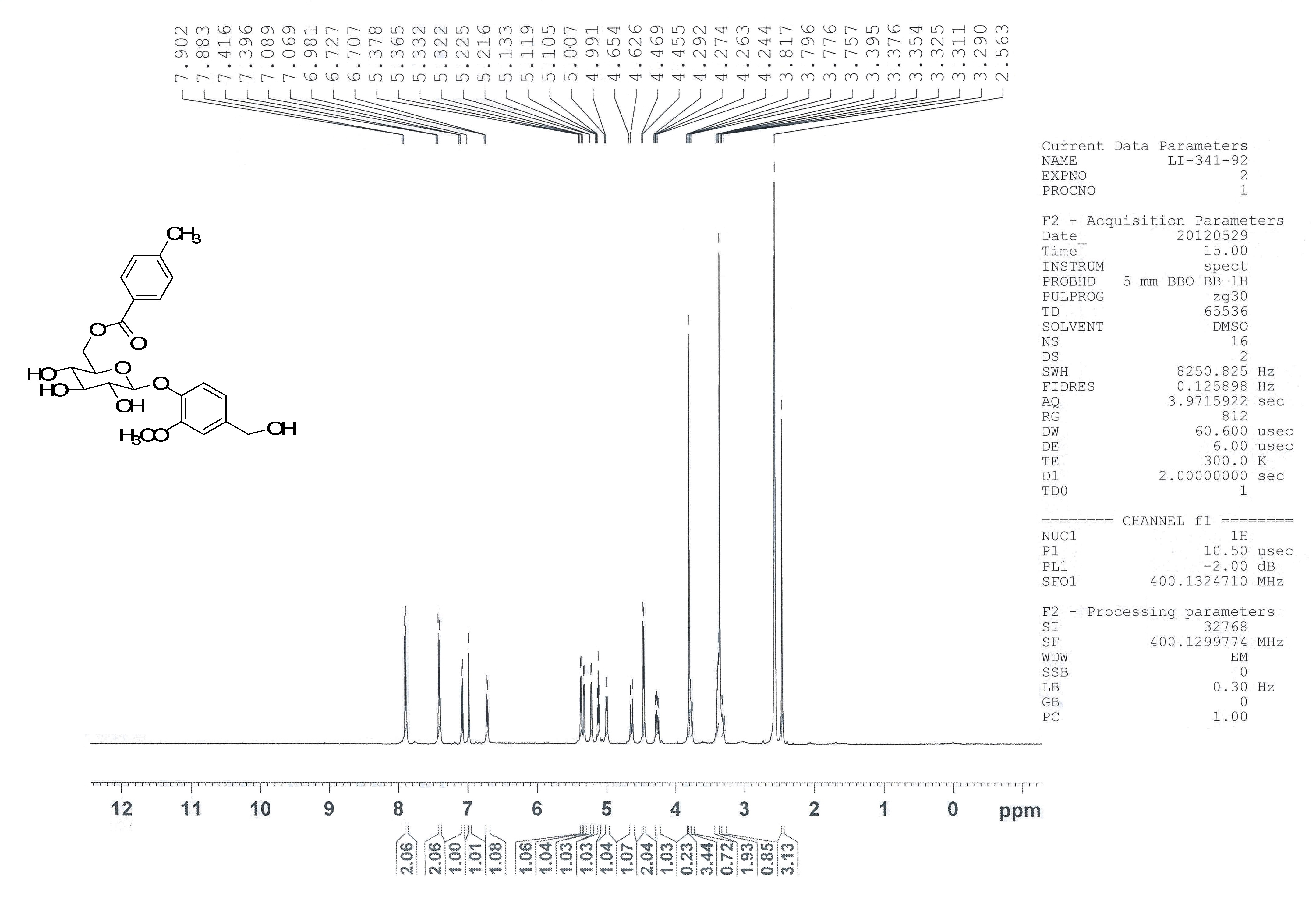** |
| **Figure 3. ^1^H NMR spectrum of compound-9c** |
| **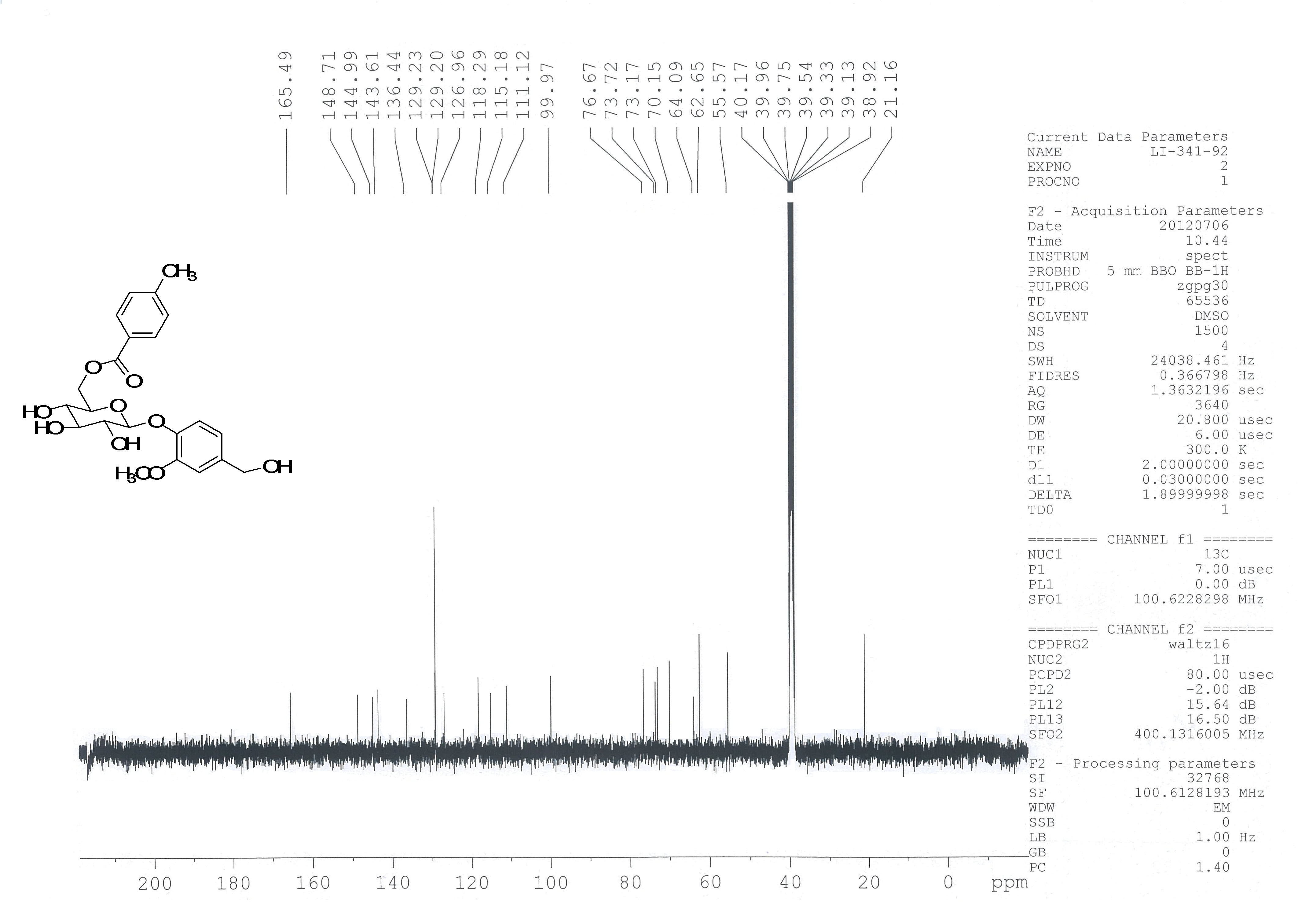** |
| **Figure 4. ^13^C NMR of spectrum of compound-9c** |
| **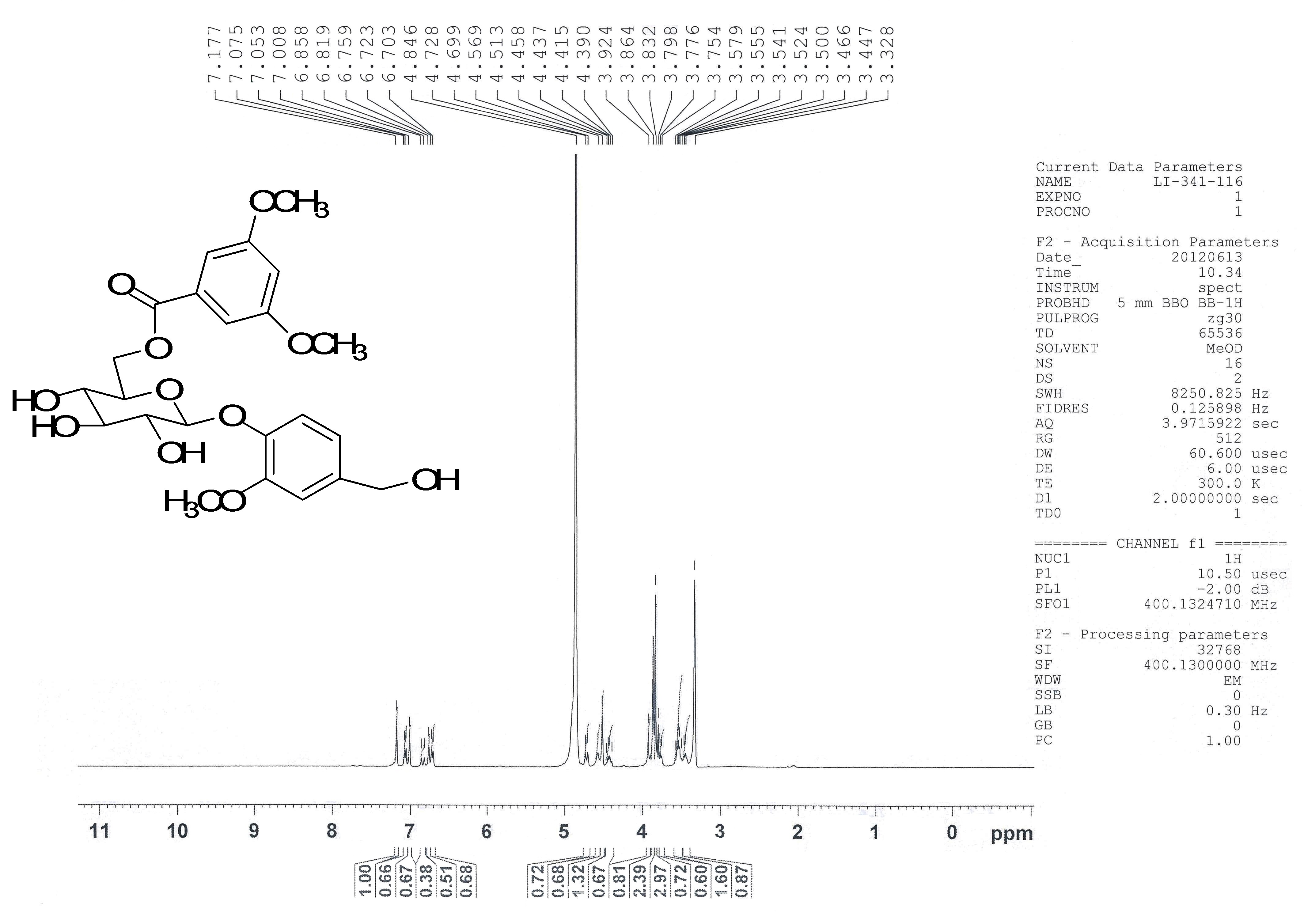** |
| **Figure 5. ^1^H NMR spectrum of compound-9d** |
| **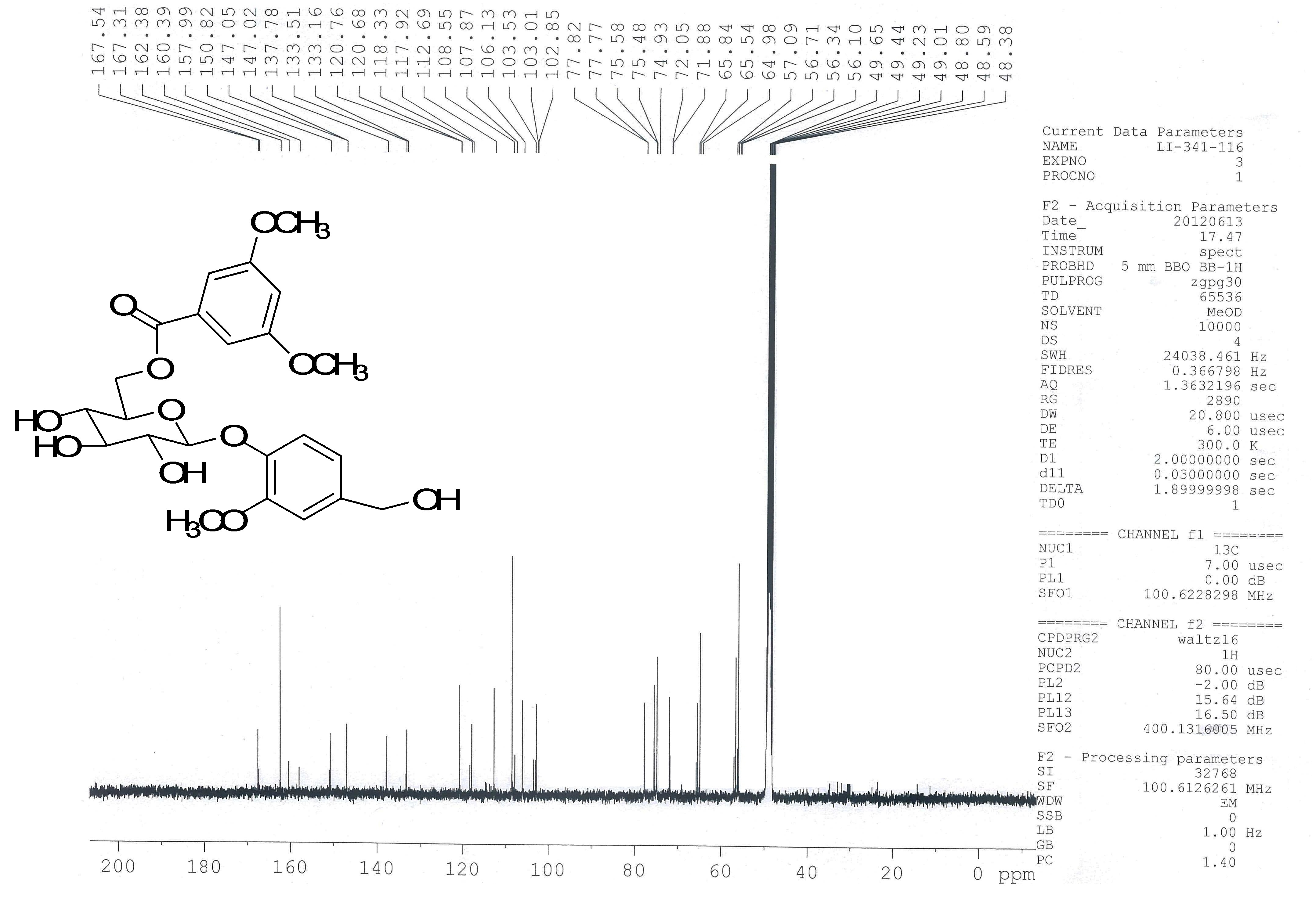** |
| **Figure 6. ^13^C NMR spectrum of compound-9d** |
| **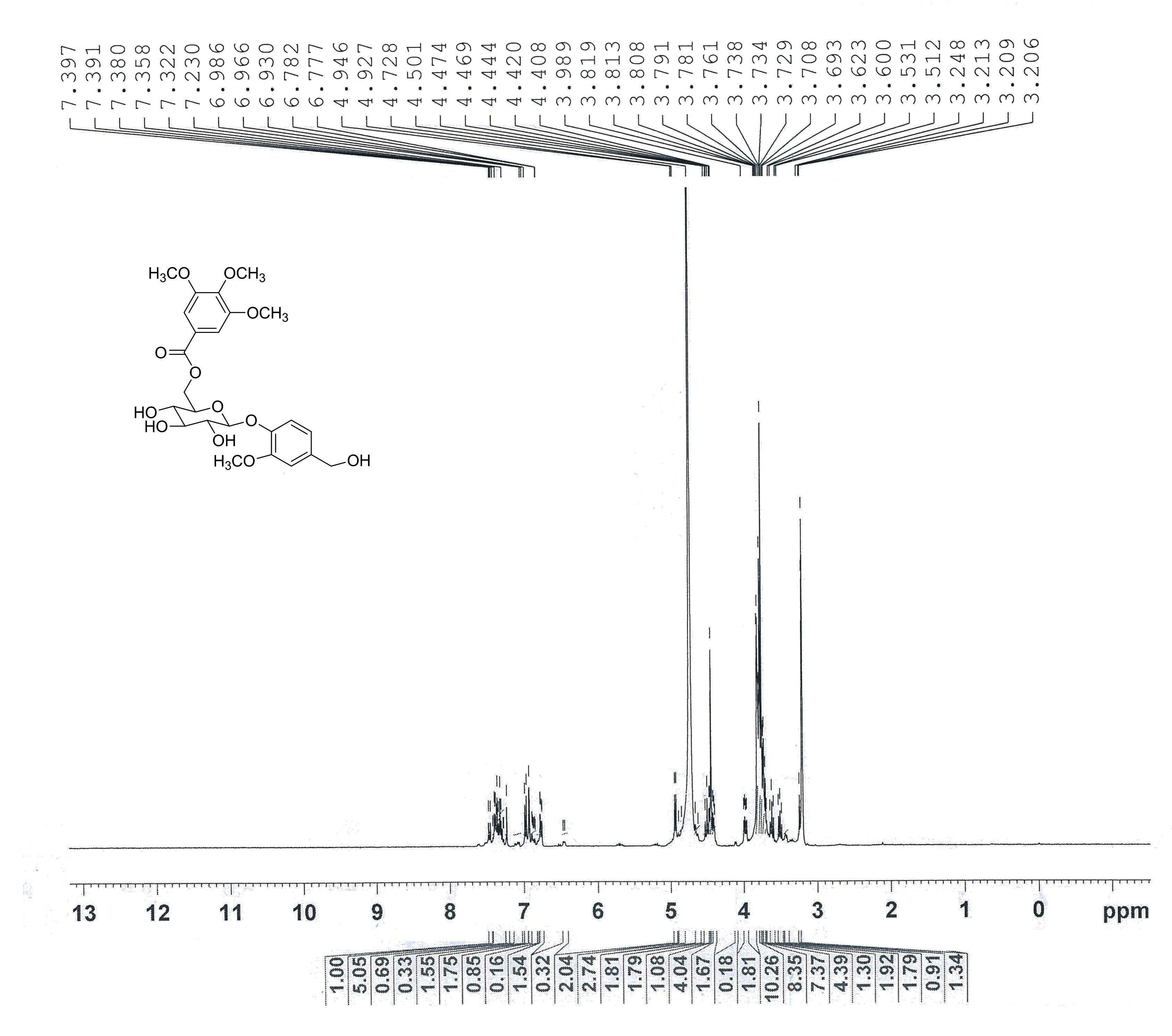** |
| **Figure 7. ^1^H NMR spectrum of compound-9e** |
| **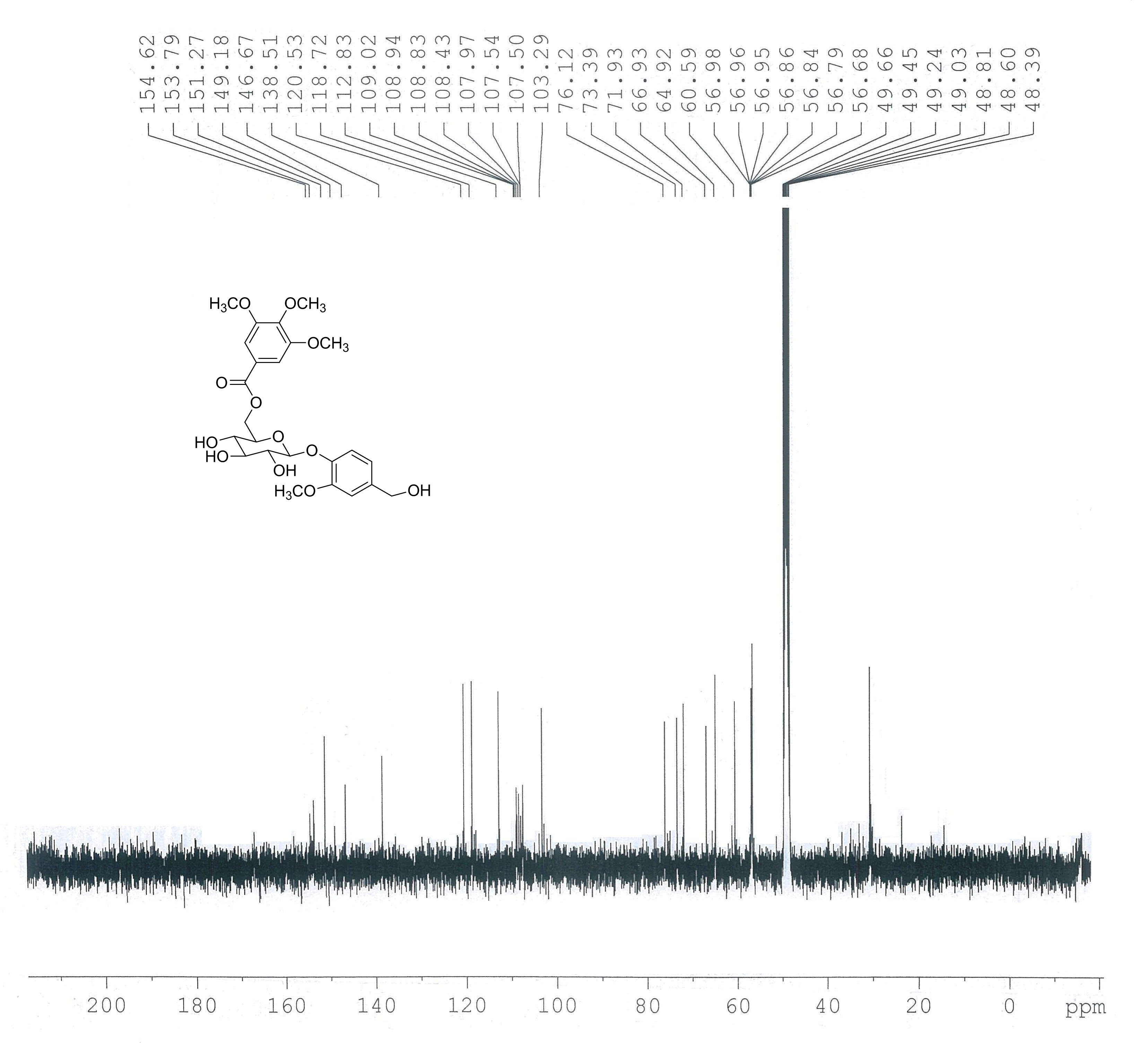** |
| **Figure 8. ^13^C NMR spectrum of compound-9e** |
| **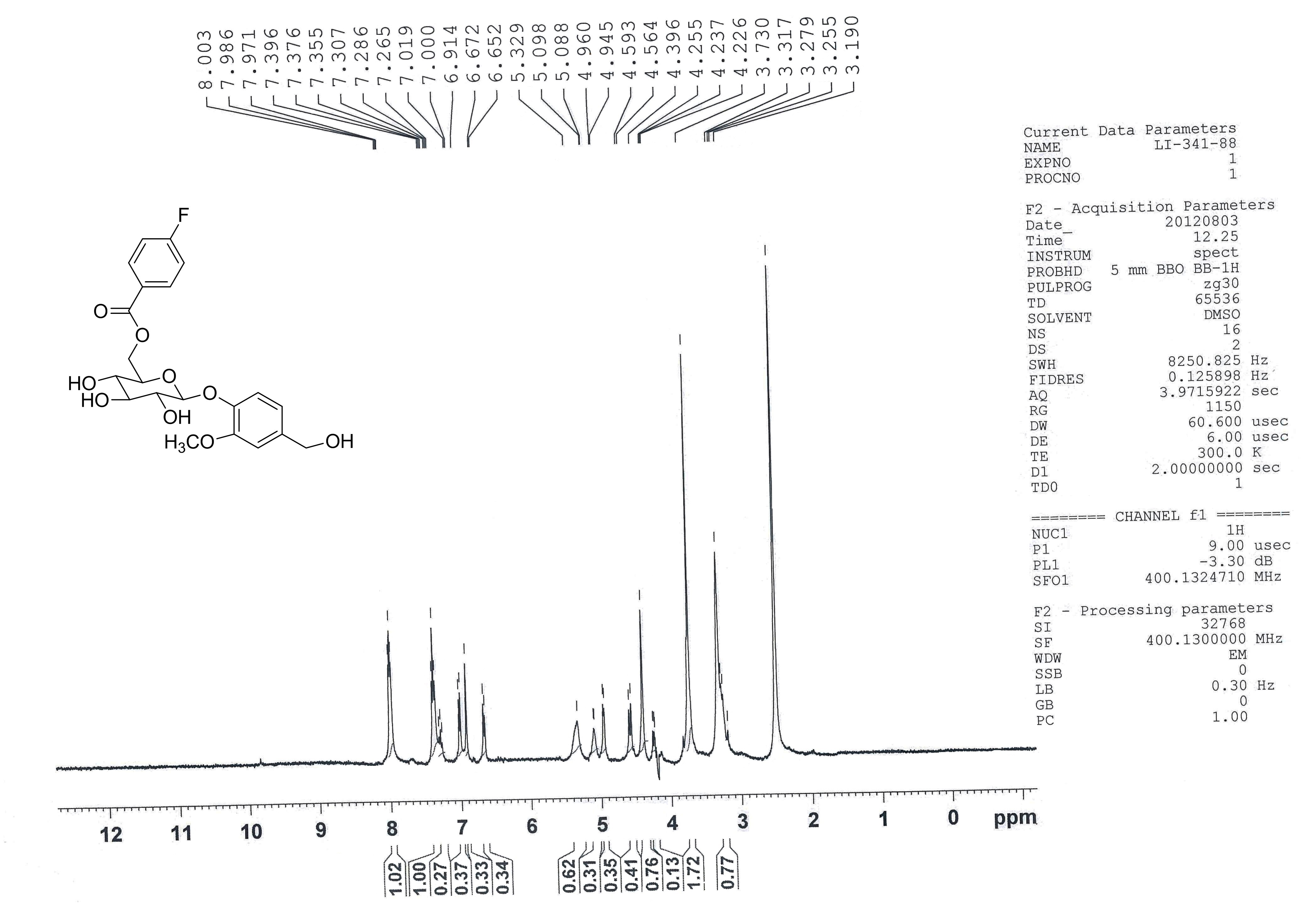** |
| **Figure 9. ^1^H NMR spectrum of compound-9f** |
| **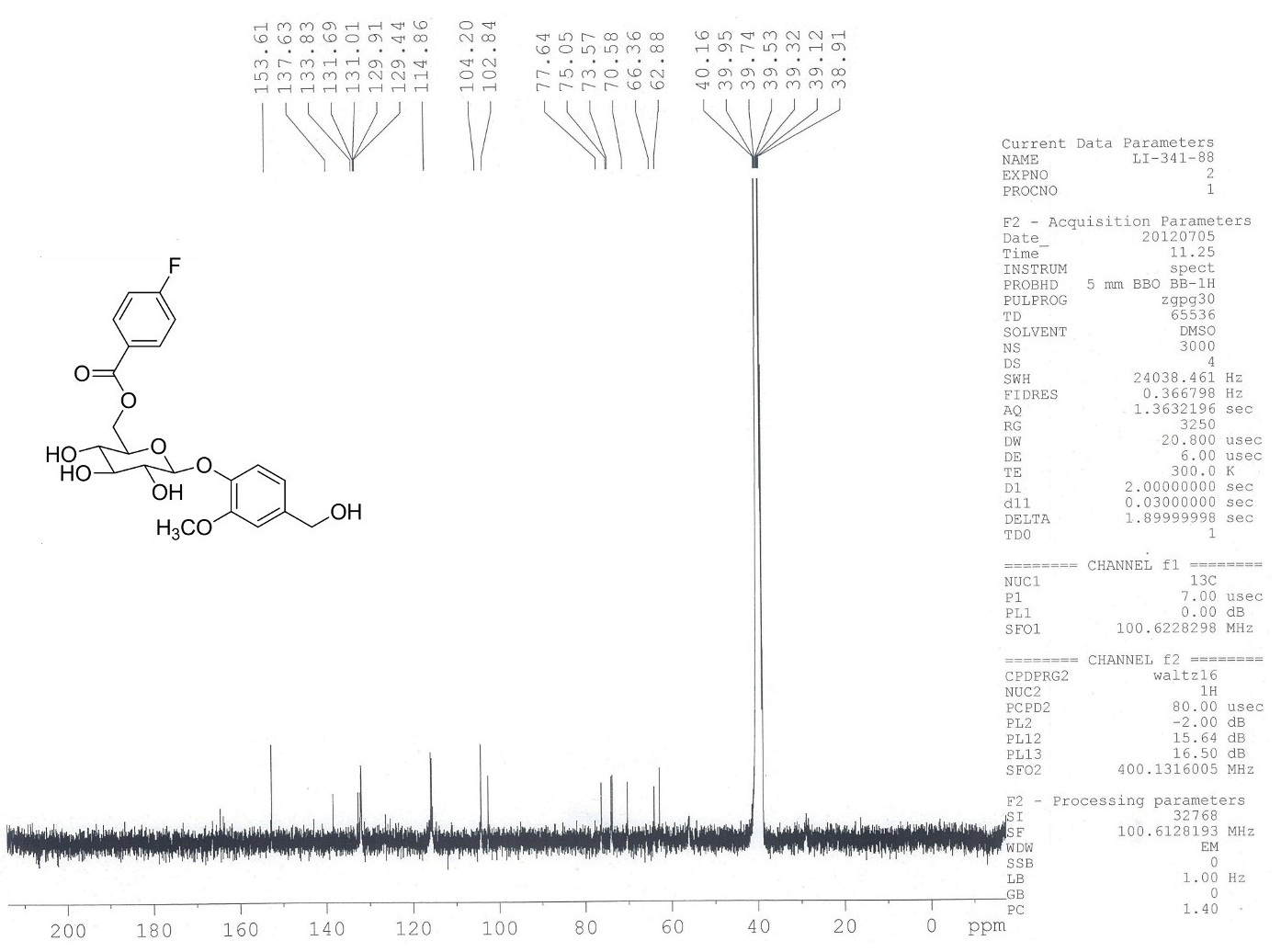** |
| **Figure 10. ^13^C NMR spectrum of compound-9f** |
| **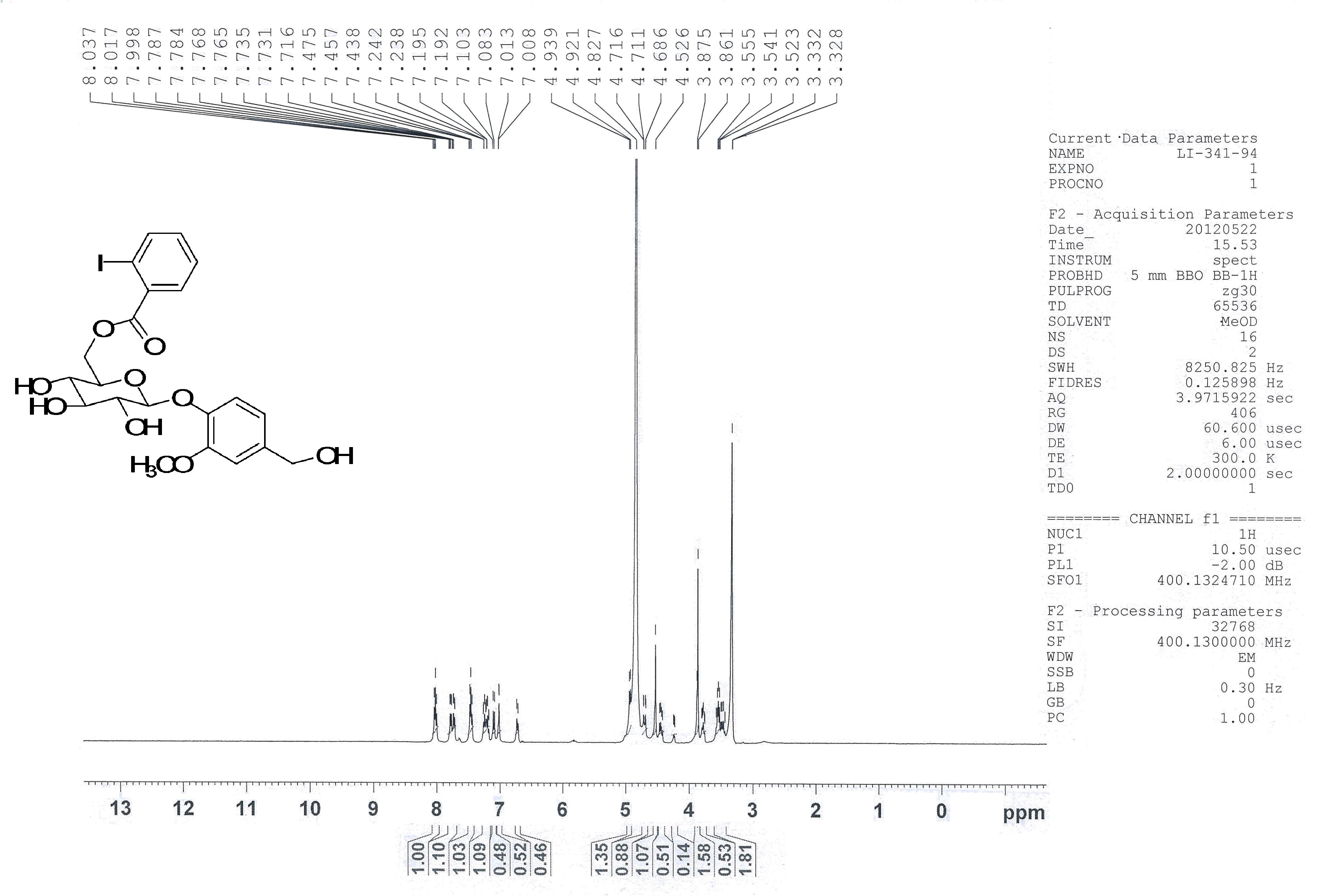** |
| **Figure 11. ^1^H NMR spectrum of compound-9g** |
| **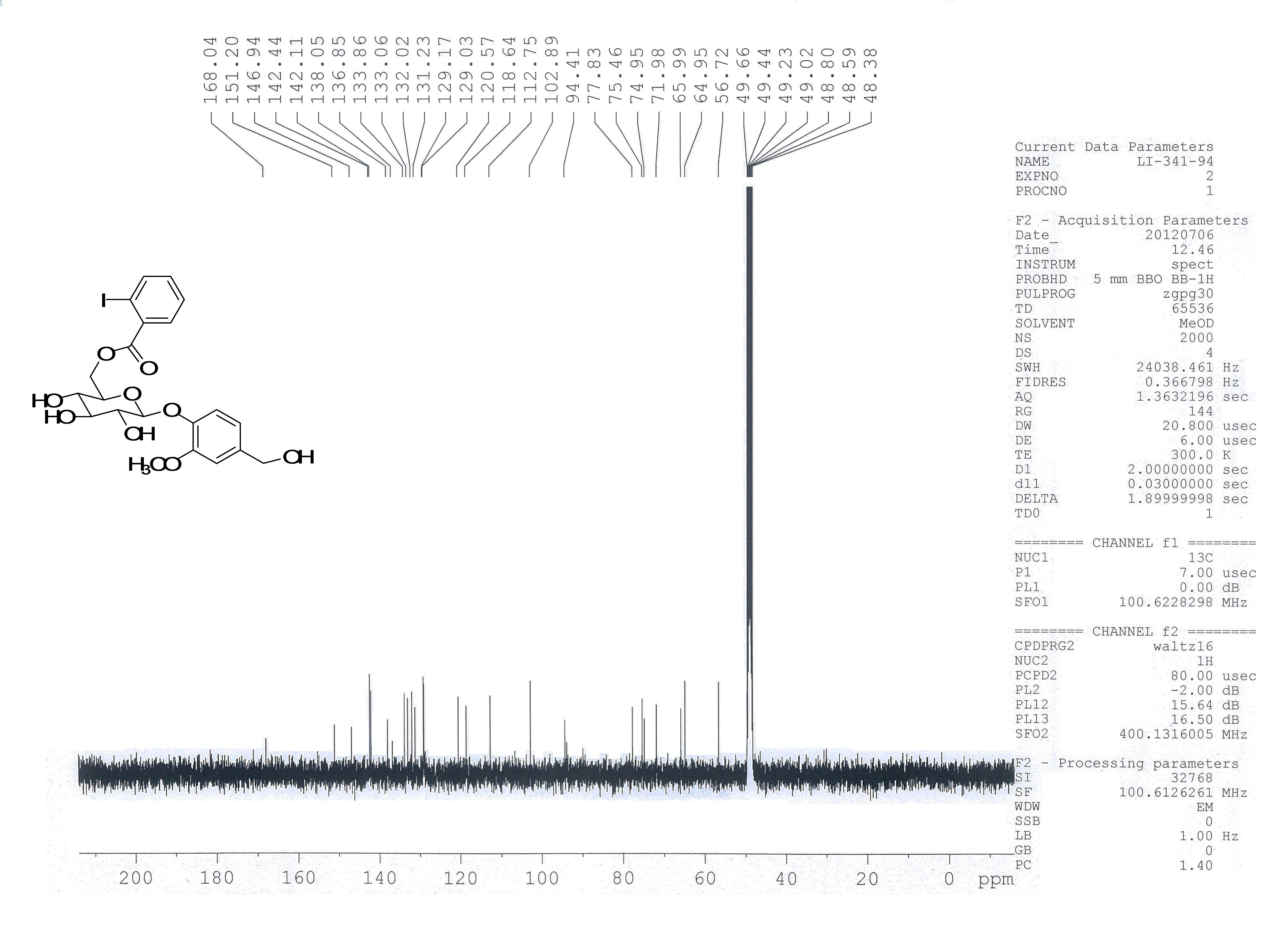** |
| **Figure 12. ^13^C NMR spectrum of compound-9g** |
| **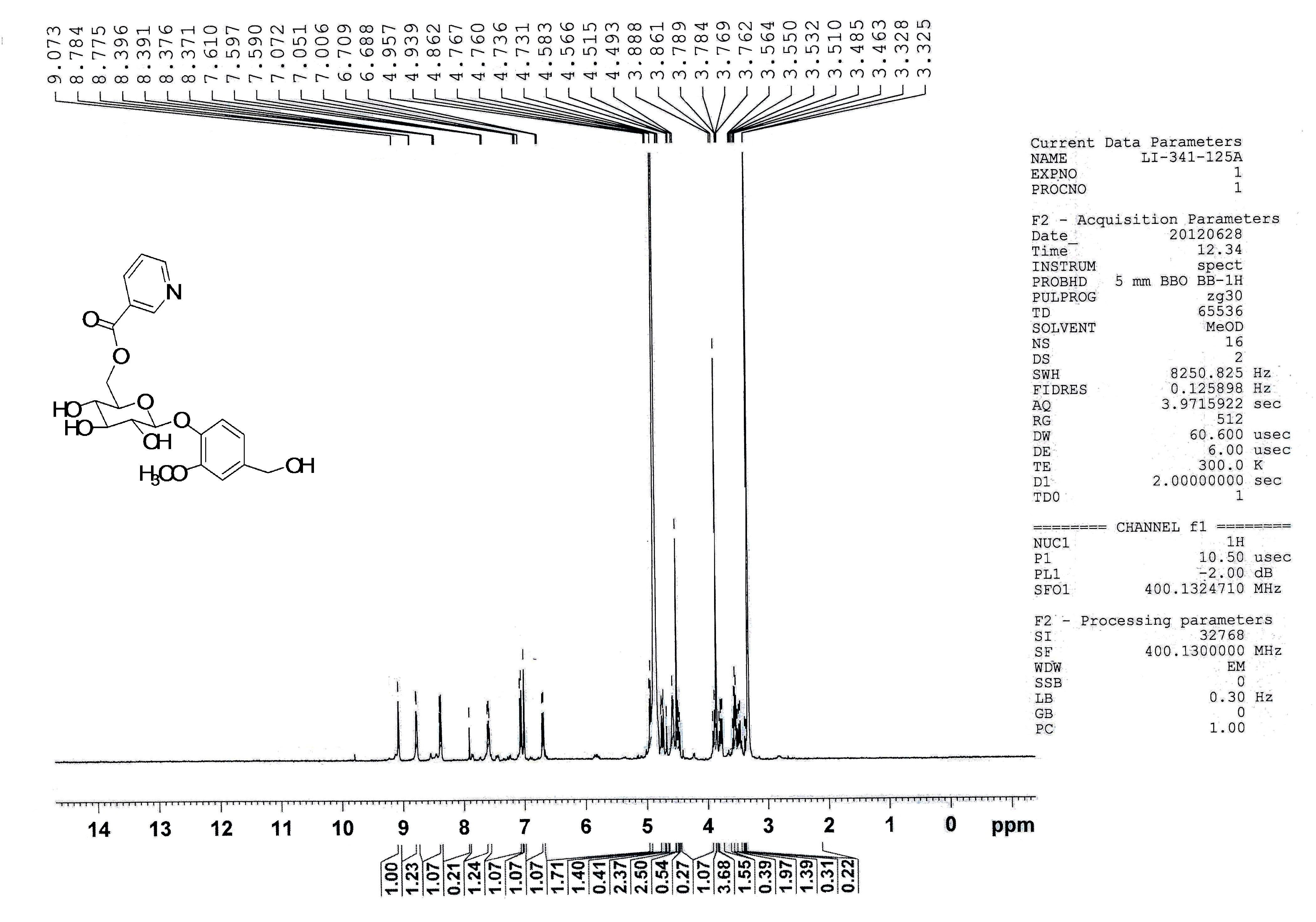** |
| **Figure 13. ^1^H NMR spectrum of compound-9h** |
| **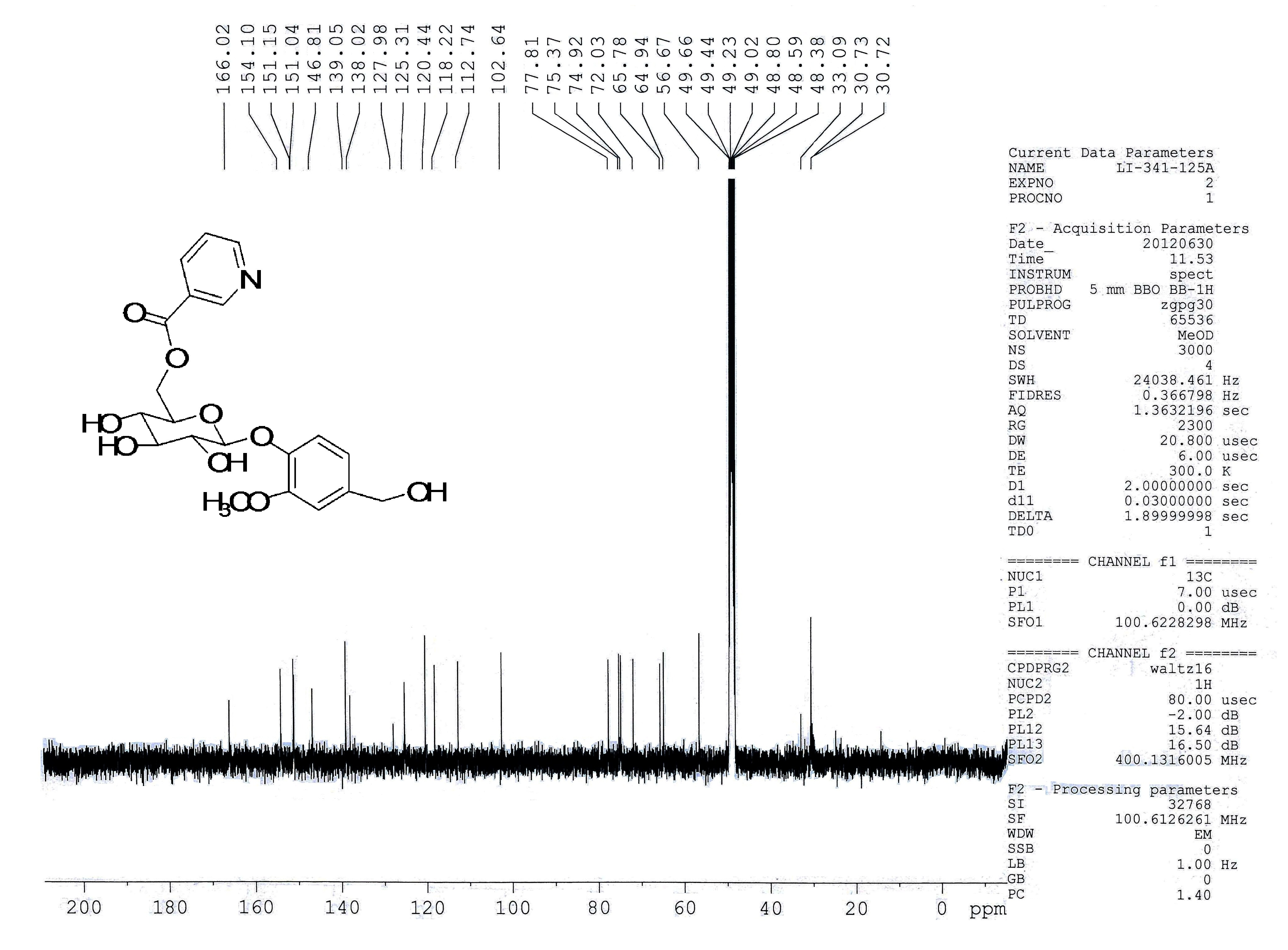** |
| **Figure 14. ^13^C NMR spectrum of compound-9h** |
| **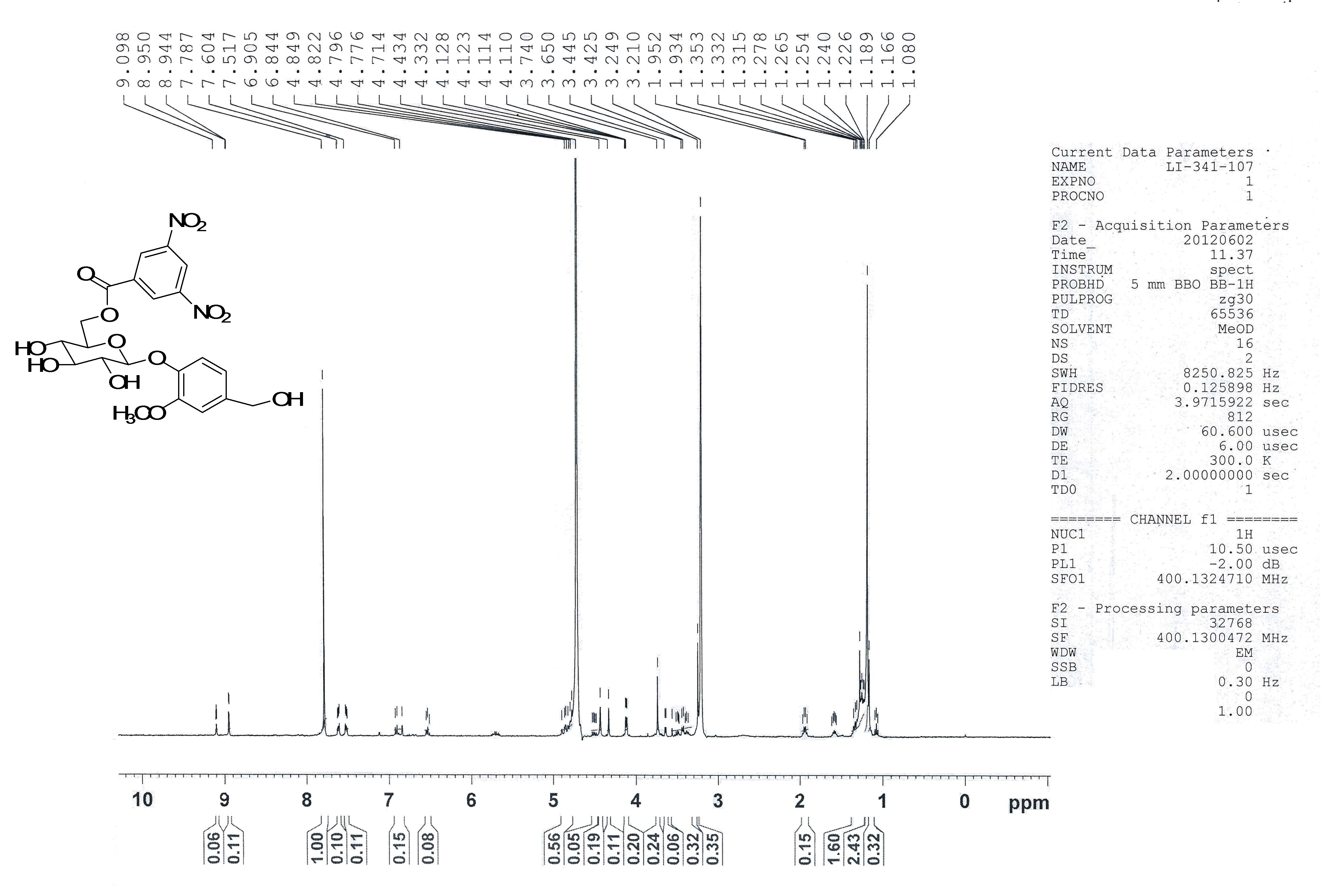** |
| **Figure 15. ^1^H NMR spectrum of compound-9i** |
| **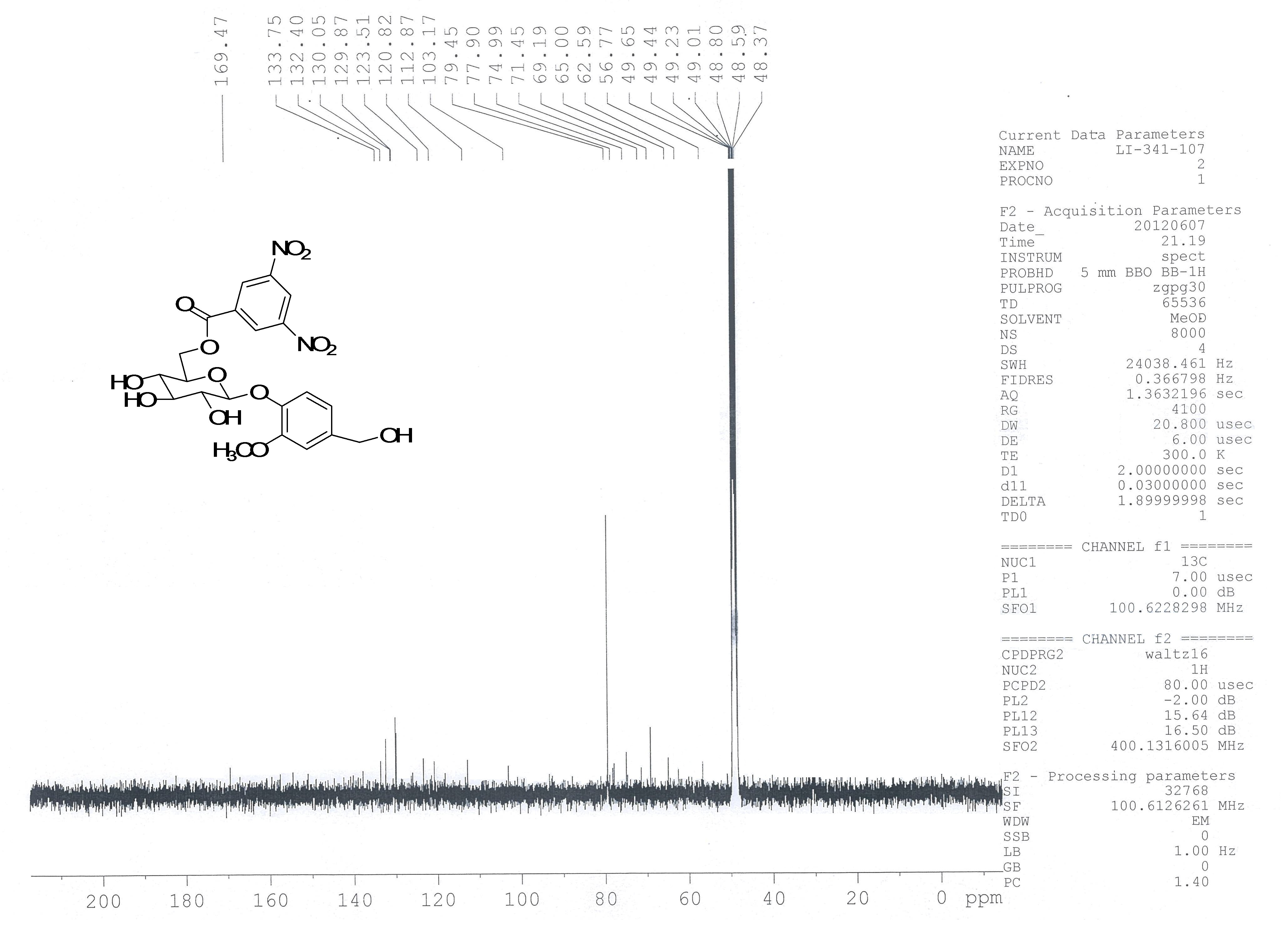** |
| **Figue 16. ^13^C NMR spectrum of compound-9i** |
| **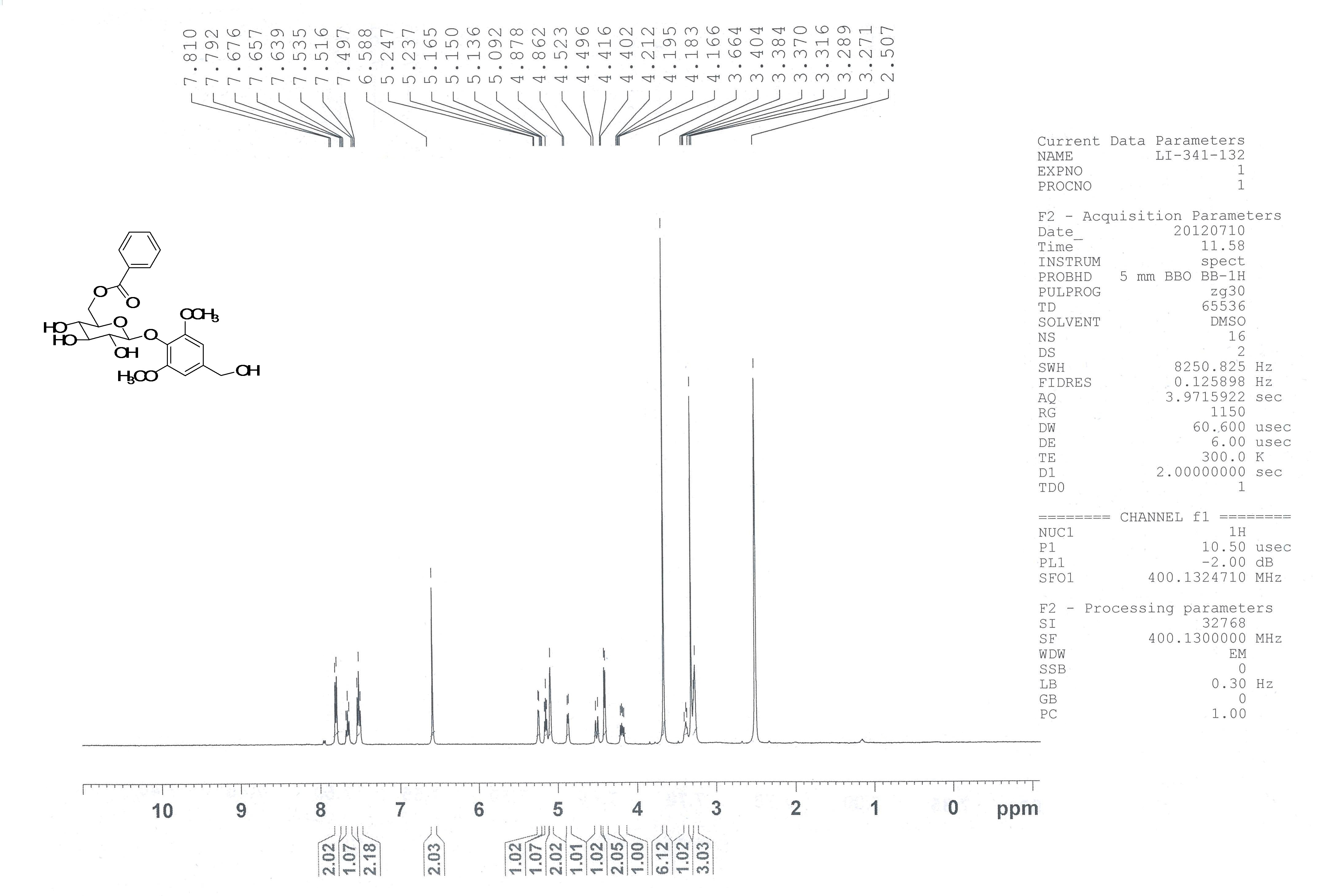** |
| **Figure 17. ^1^H NMR spectrum of compound-9j** |
| **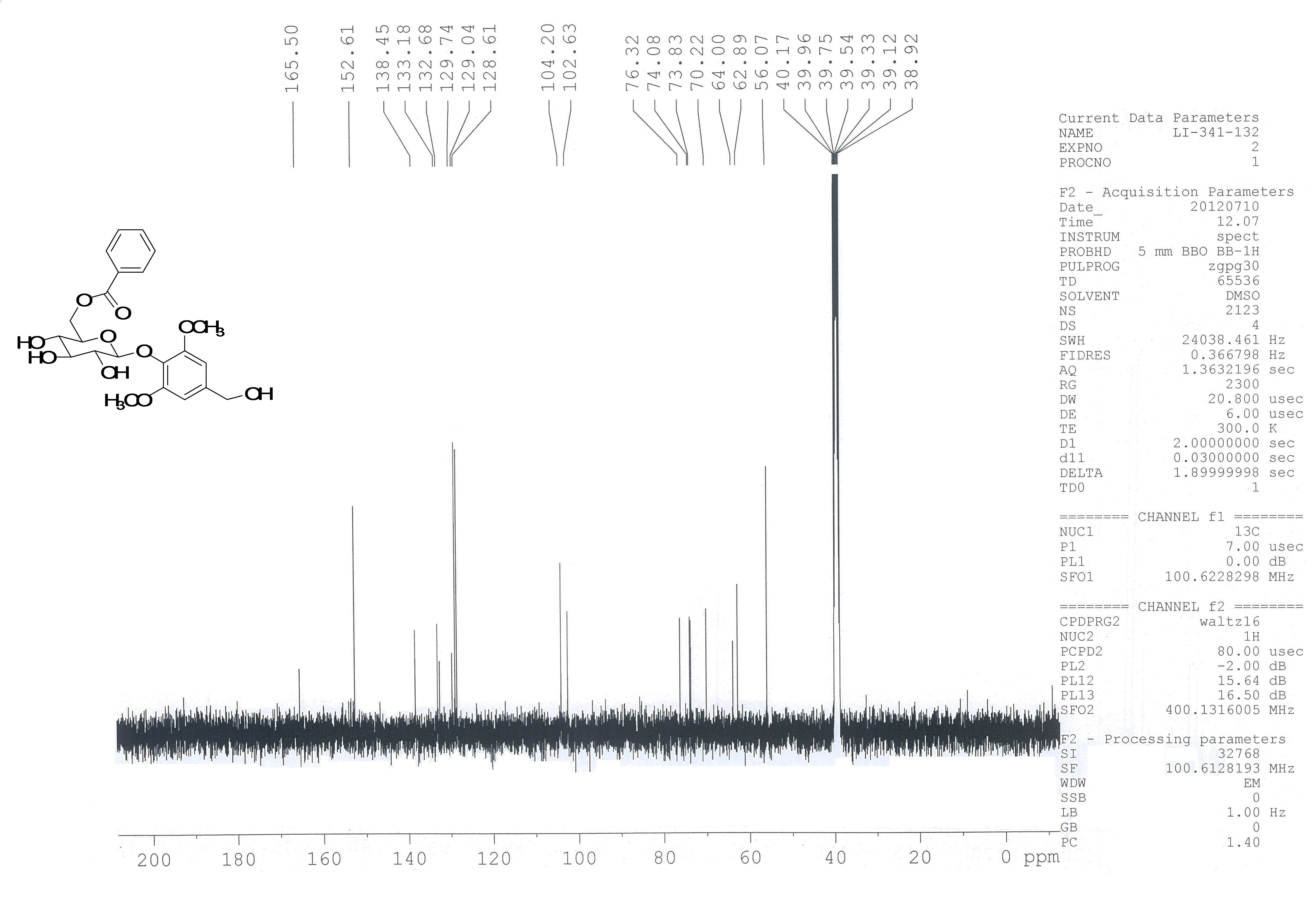** |
| **Figure 18. ^13^C NMR spectrum of compound-9j** |
| **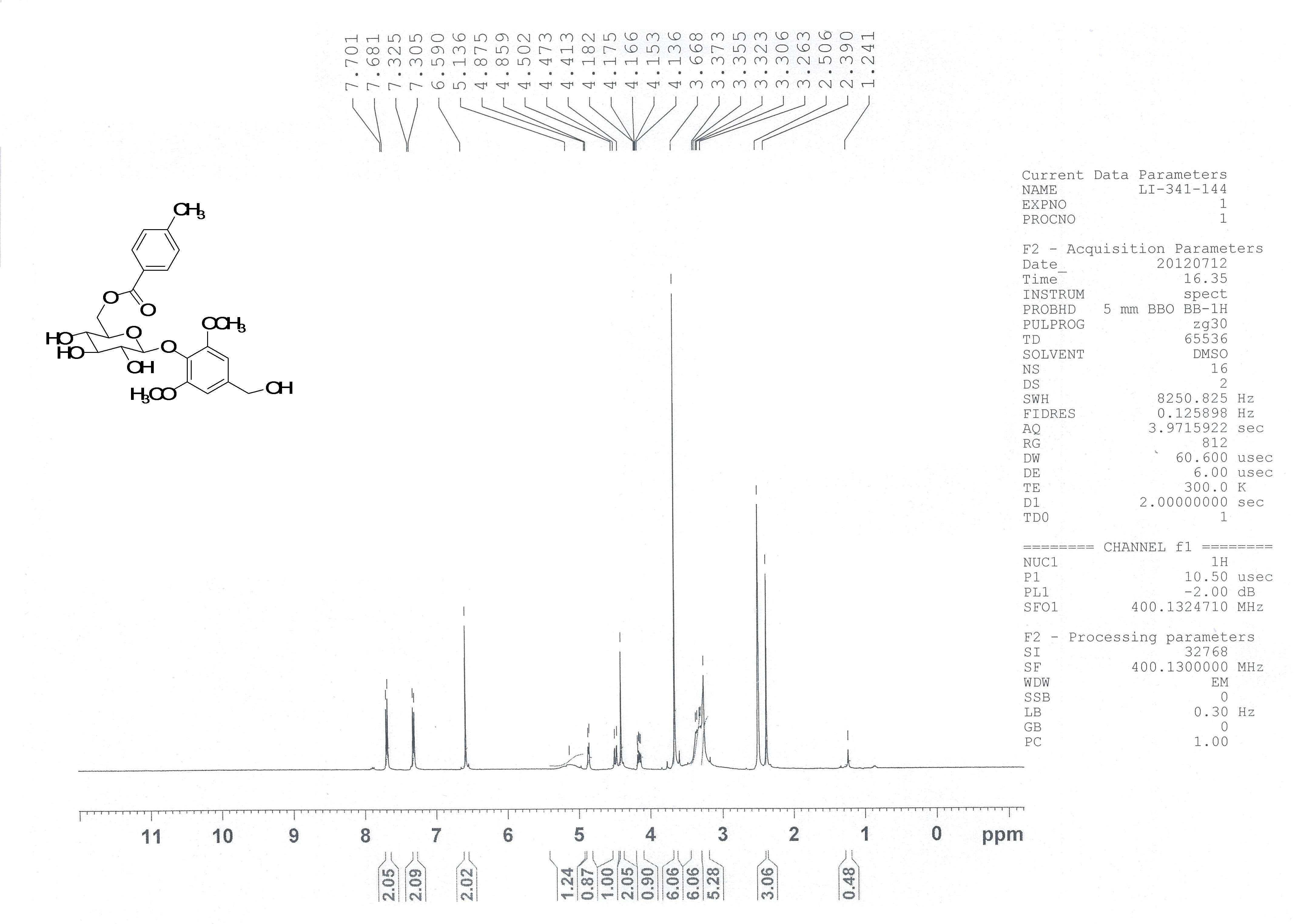** |
| **Figure 19. ^1^H NMR spectrum of compound-9k** |
| **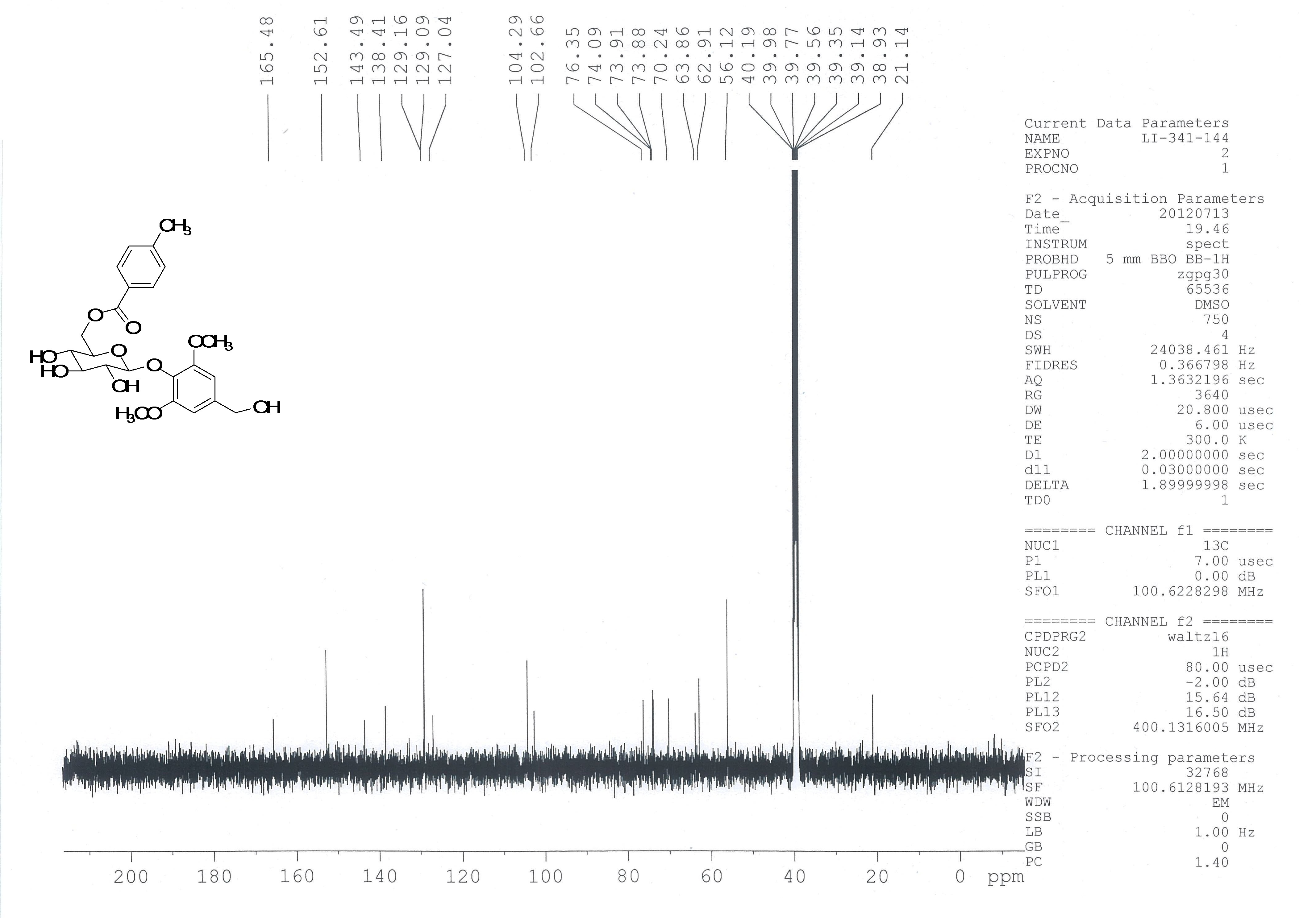** |
| **Figure 20. ^13^C NMR spectrum of compound-9k** |
| **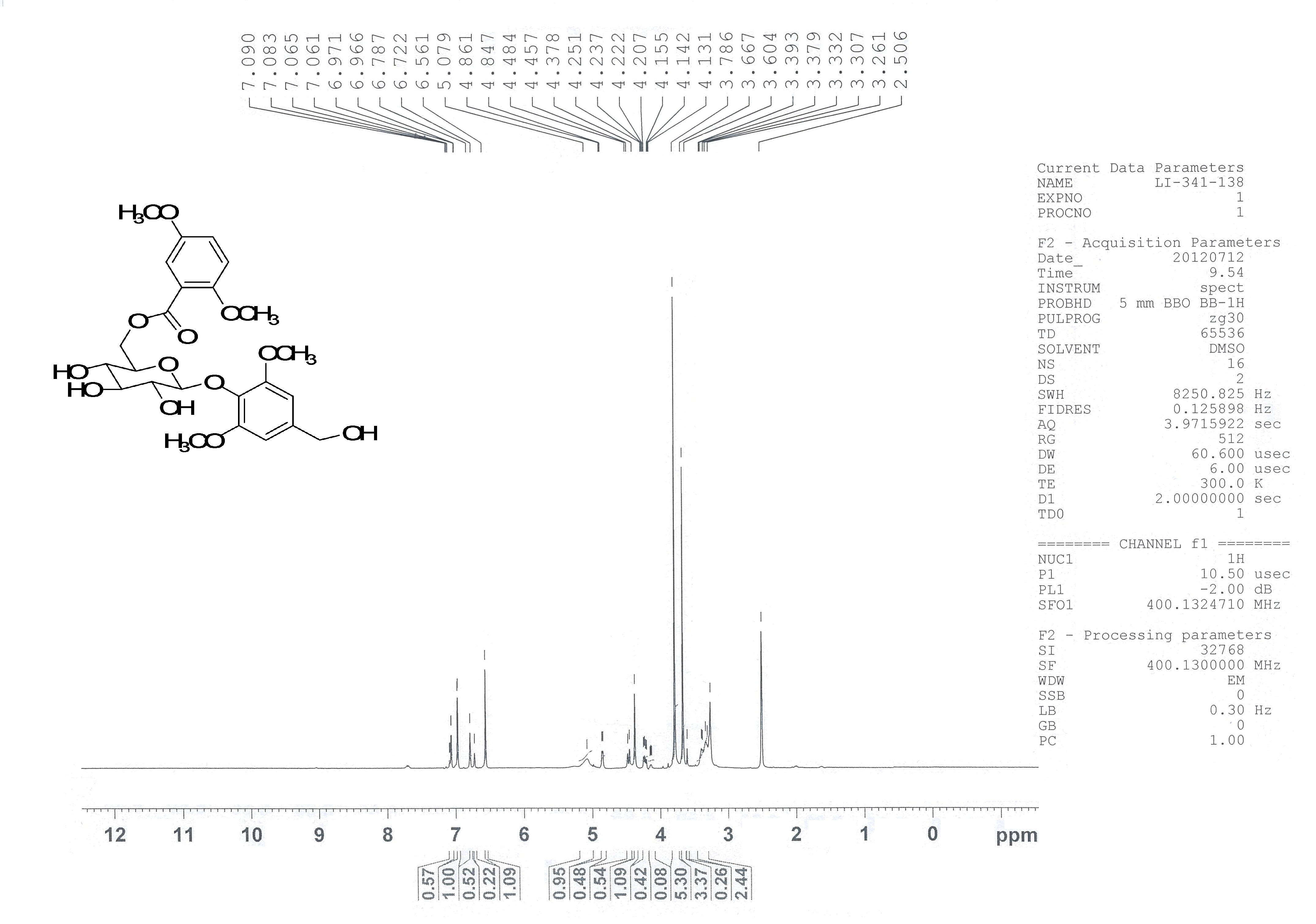** |
| **Figure 21. ^1^H NMR spectrum of compound-9l** |
| **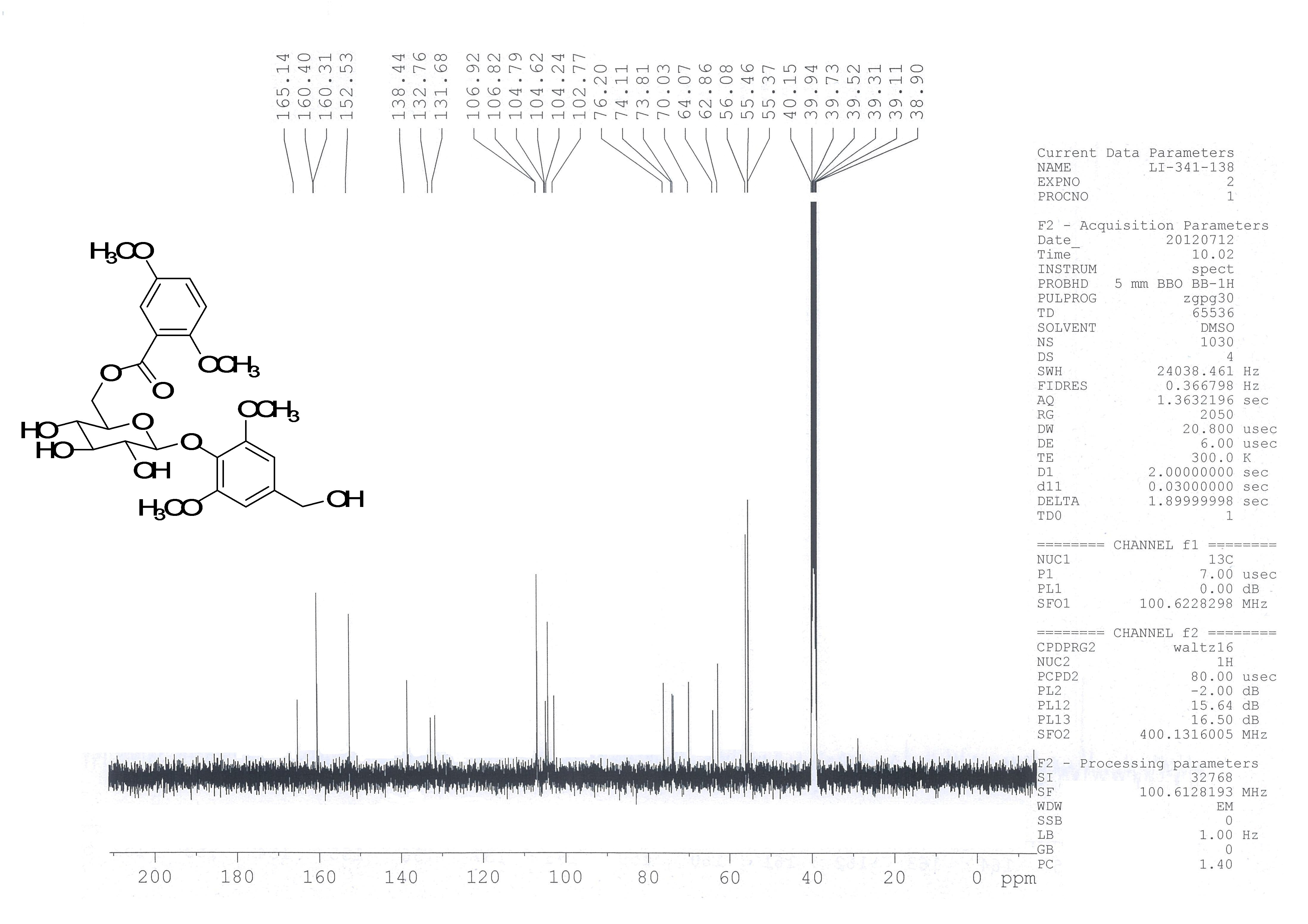** |
| **Figure 22. ^13^C NMR spectrum of compound-9l** |
| **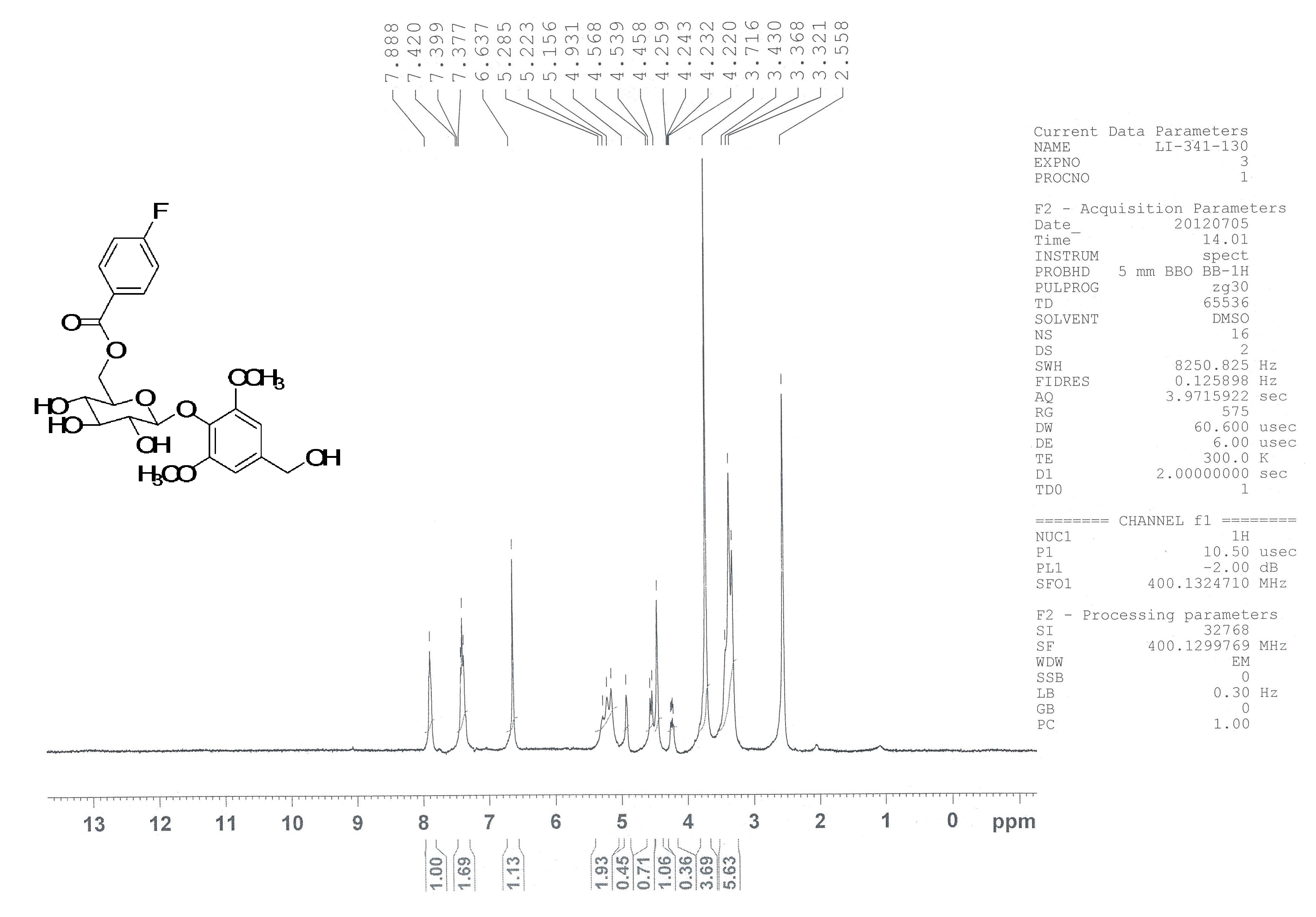** |
| **Figure 23. ^1^H NMR spectrum of compound-9m** |
| **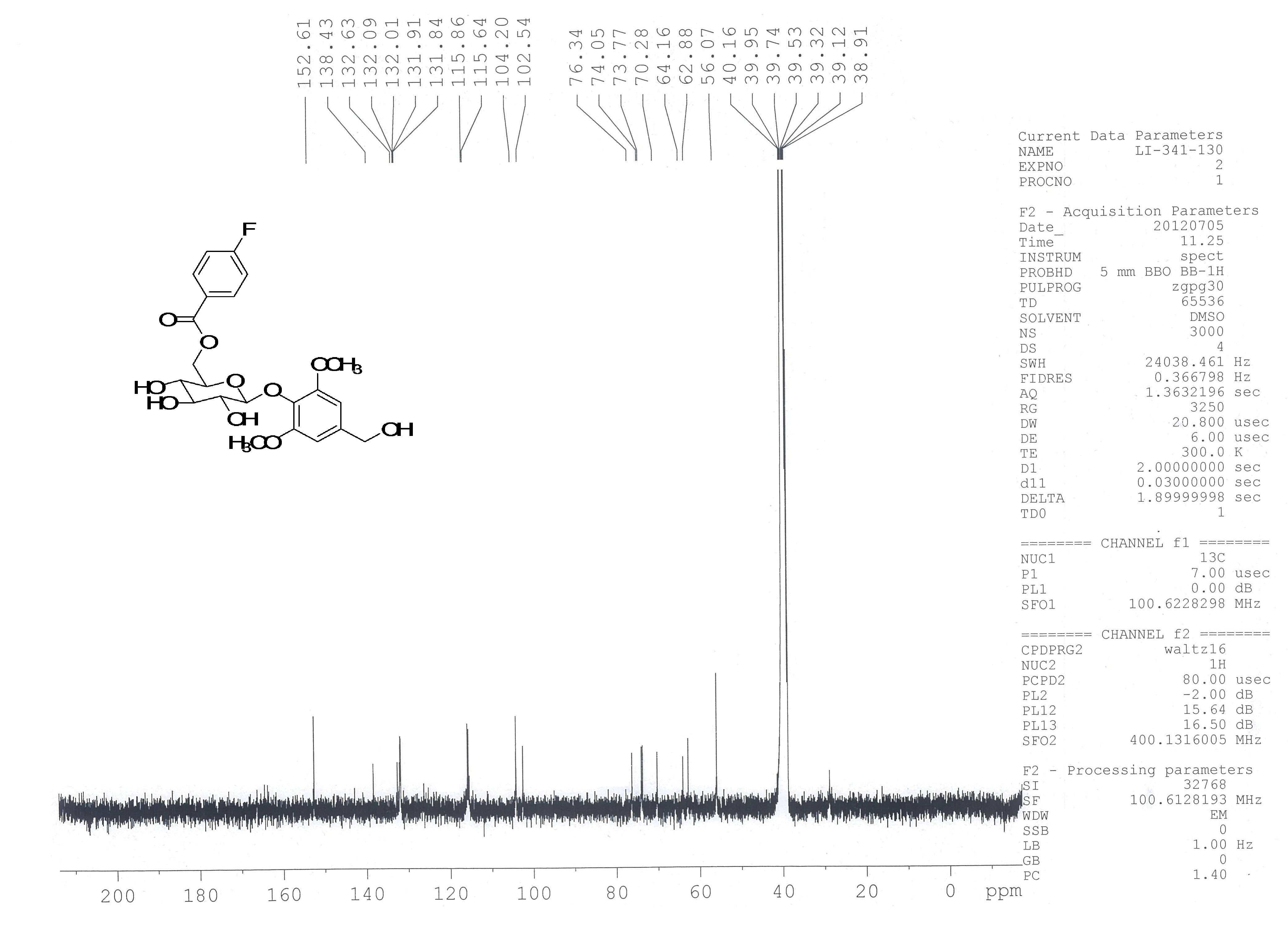** |
| **Figure 24. ^13^C NMR spectrum of compound-9m** |
| **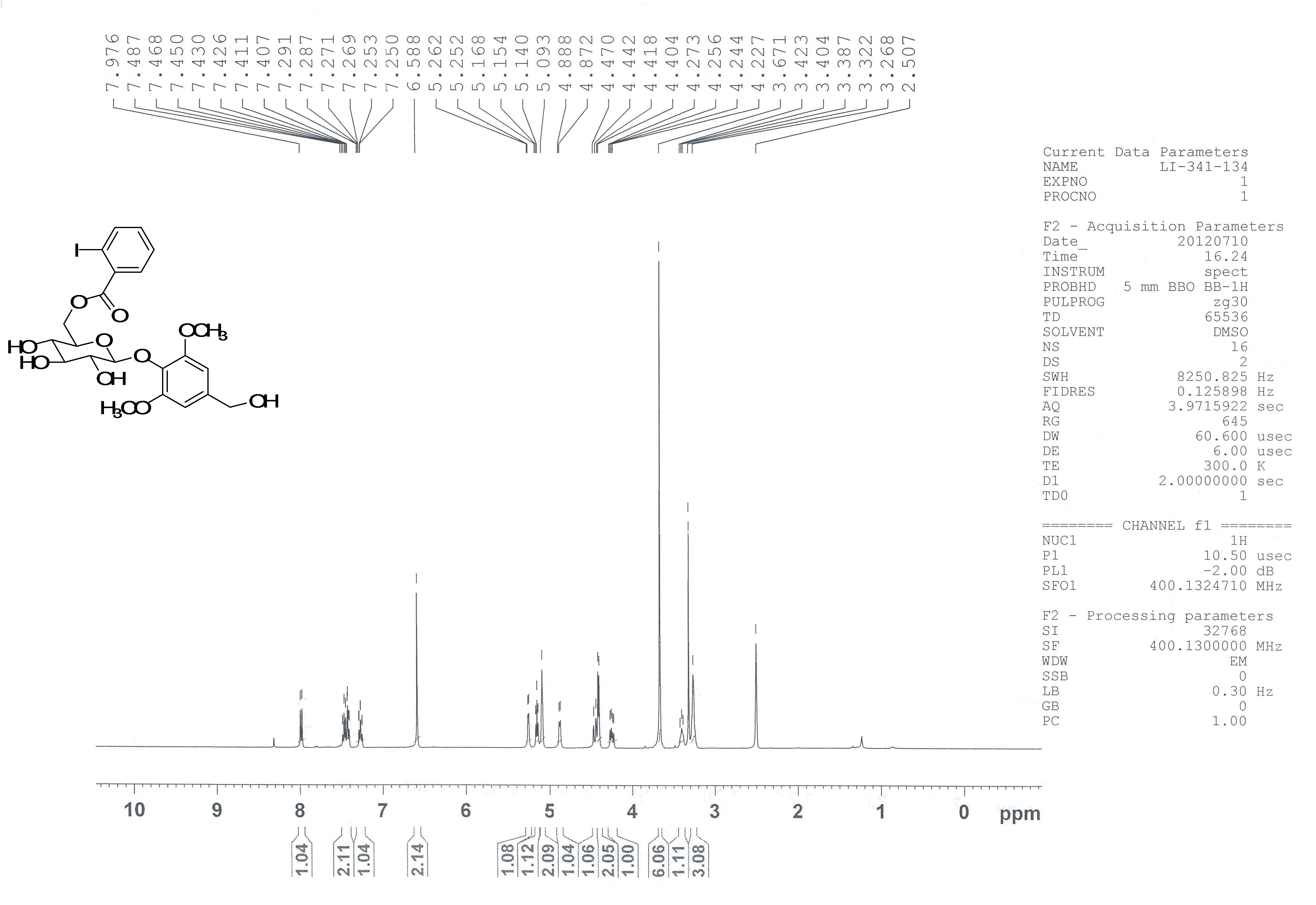** |
| **Figure 25. ^1^H NMR spectrum of compound-9n** |
| **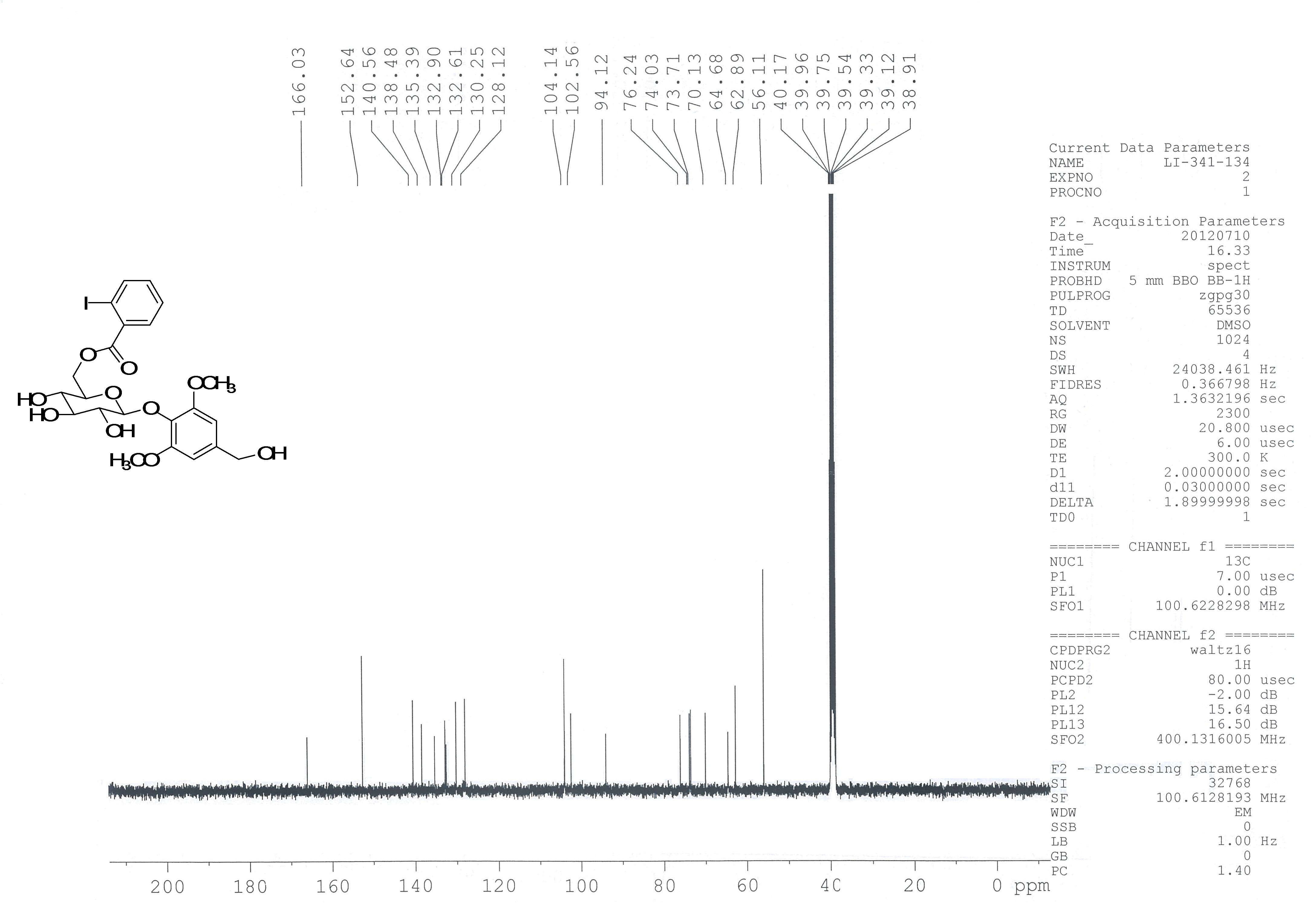** |
| **Figure 26. ^13^C NMR spectrum of compound-9n** |
| **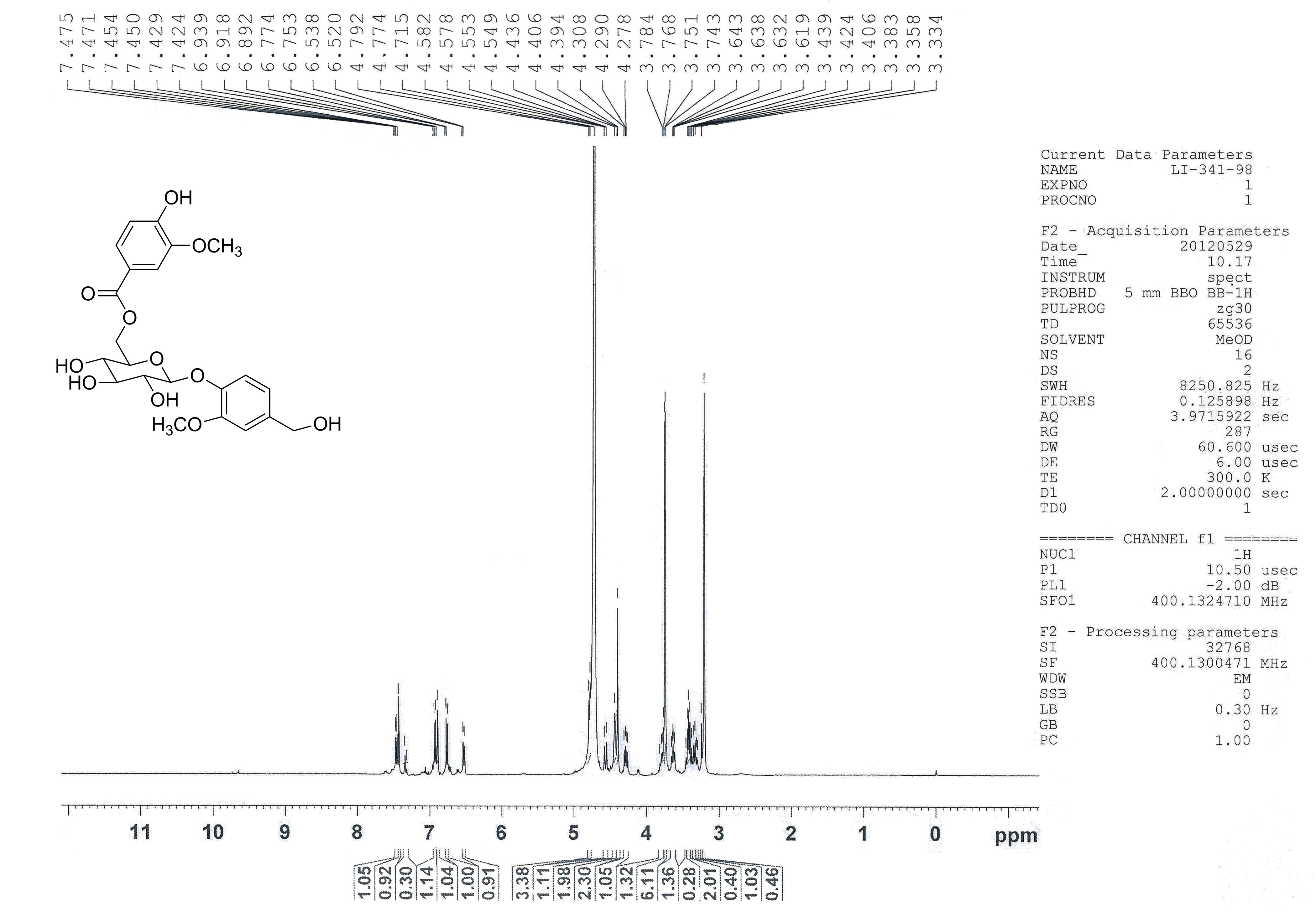** |
| **Figure 27. ^1^H NMR spectrum of saccharumoside-B (10)** |
| **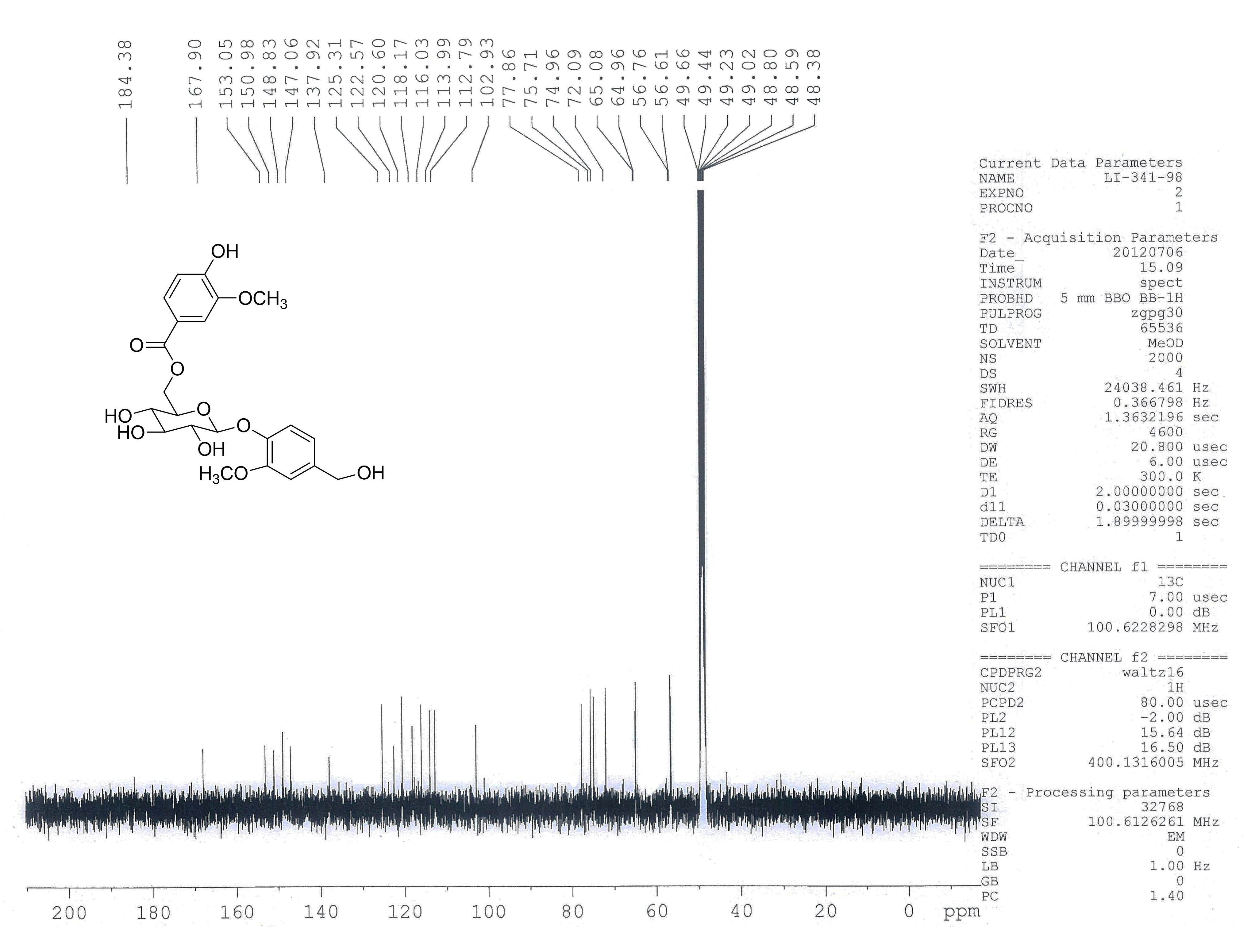** |

**Figure 28. ^13^C NMR spectrum of saccharumoside-B (10)**

A
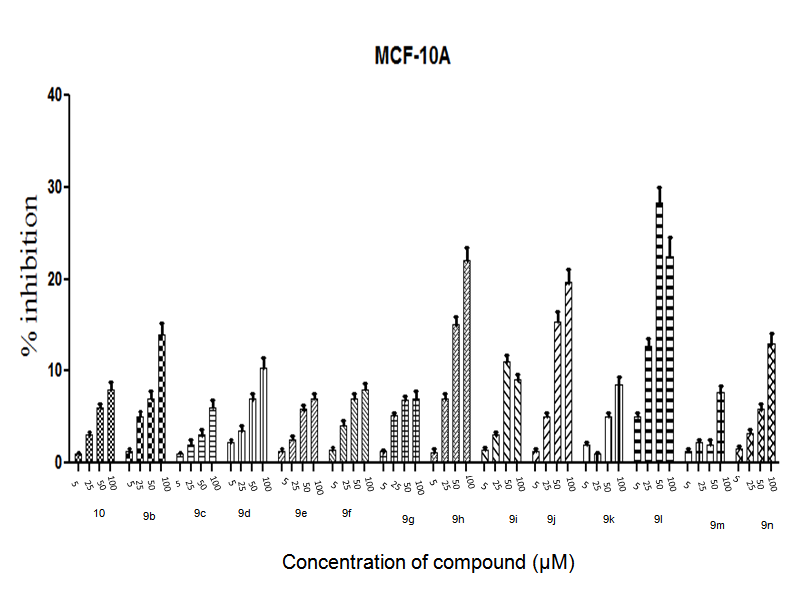


B
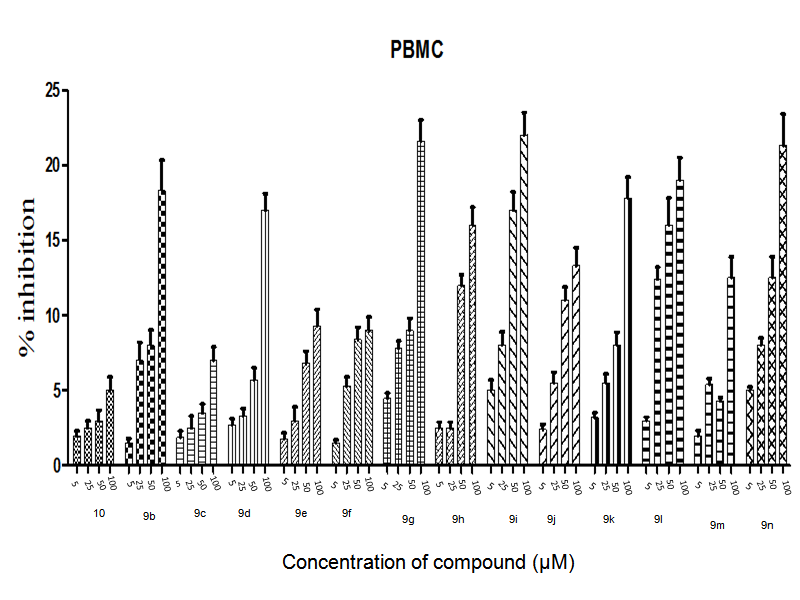


**Figure** **29**. **Antiproliferative effect of compounds (10, 9a-9n) on normal cells.** Cells were treated with compounds for 48 h and proliferation was assessed by MTT assay. (A) Antiproliferative effect of compounds on MCF10A cells. (B) Antiproliferative effect of compounds on PBMC cells.


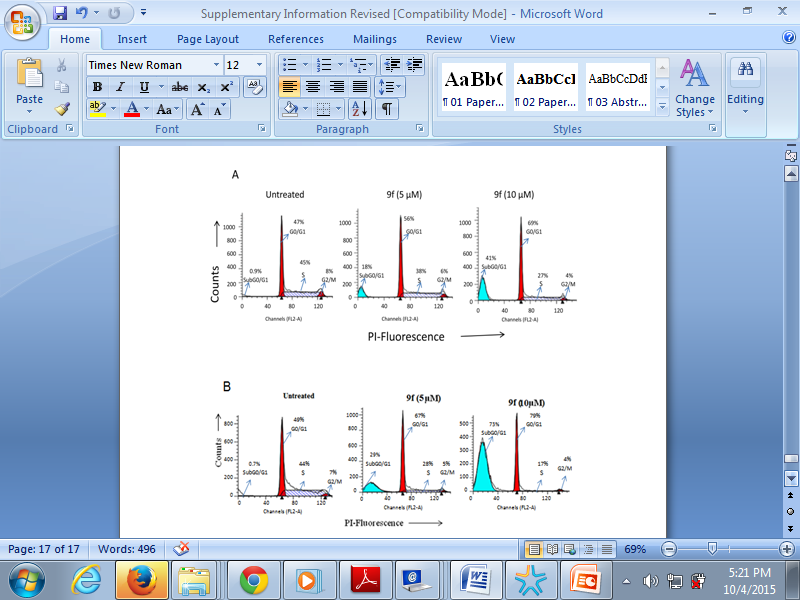


**Figure 30**. **Effect of 9f on Cell cycle of cancer cells was analyzed by flow cytometry and represented in histograms.** (A) Effect of **9f** on Cell cycle of MCF-7 breast cancer cells. (B) Effect of **9f** on cell cycle of HL-60 leukemia cells.


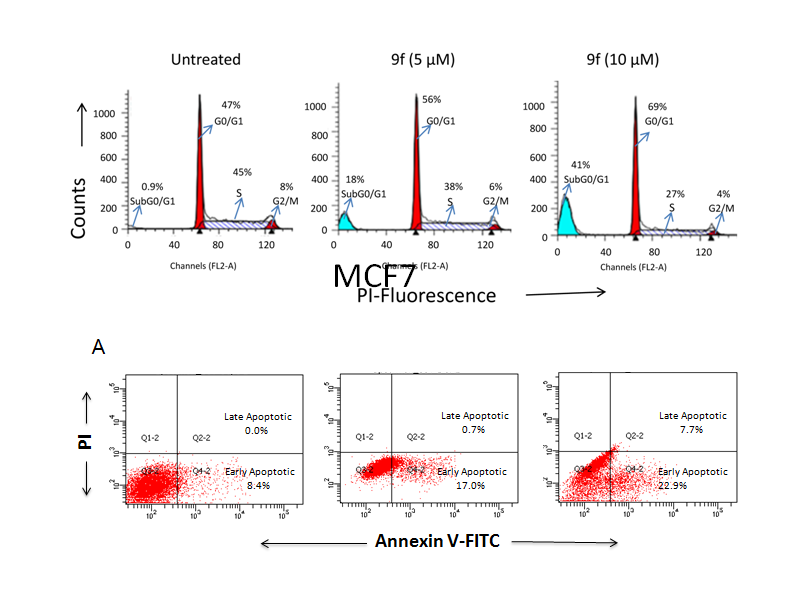


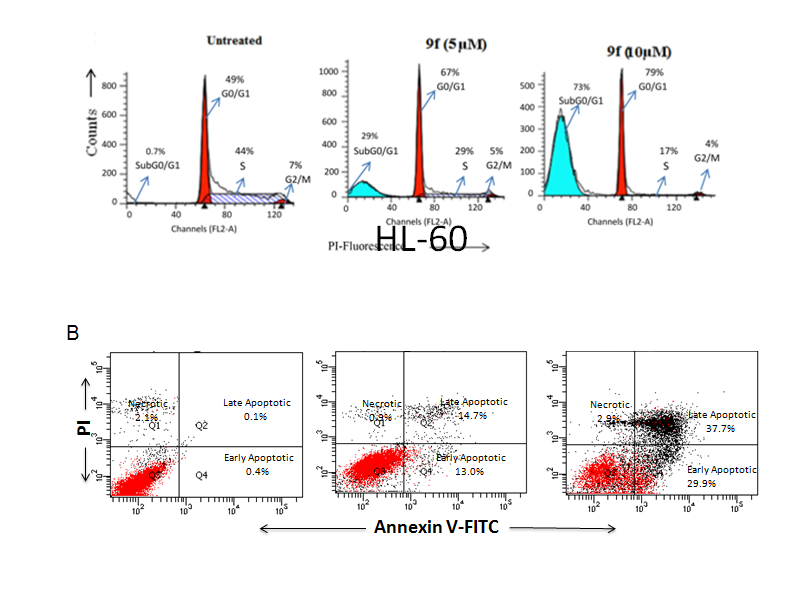


**Figure 31**. **Pro-apoptotic effect of 9f on cancer cells was analyzed by flow cytometry and represented in histograms.** (A) Pro-apoptotic effect of **9f** on MCF-7 breast cancer cells. (B) Pro-apoptotic effect of **9f** on HL-60 leukemia cells.
